# Disease severity-specific neutrophil signatures in blood transcriptomes stratify COVID-19 patients

**DOI:** 10.1186/s13073-020-00823-5

**Published:** 2021-01-13

**Authors:** Anna C. Aschenbrenner, Maria Mouktaroudi, Benjamin Krämer, Marie Oestreich, Nikolaos Antonakos, Melanie Nuesch-Germano, Konstantina Gkizeli, Lorenzo Bonaguro, Nico Reusch, Kevin Baßler, Maria Saridaki, Rainer Knoll, Tal Pecht, Theodore S. Kapellos, Sarandia Doulou, Charlotte Kröger, Miriam Herbert, Lisa Holsten, Arik Horne, Ioanna D. Gemünd, Nikoletta Rovina, Shobhit Agrawal, Kilian Dahm, Martina van Uelft, Anna Drews, Lena Lenkeit, Niklas Bruse, Jelle Gerretsen, Jannik Gierlich, Matthias Becker, Kristian Händler, Michael Kraut, Heidi Theis, Simachew Mengiste, Elena De Domenico, Jonas Schulte-Schrepping, Lea Seep, Jan Raabe, Christoph Hoffmeister, Michael ToVinh, Verena Keitel, Gereon Rieke, Valentina Talevi, Dirk Skowasch, N. Ahmad Aziz, Peter Pickkers, Frank L. van de Veerdonk, Mihai G. Netea, Joachim L. Schultze, Matthijs Kox, Monique M. B. Breteler, Jacob Nattermann, Antonia Koutsoukou, Evangelos J. Giamarellos-Bourboulis, Thomas Ulas, German COVID-19 Omics Initiative (DeCOI), Janine Altmüller, Angel Angelov, Robert Bals, Alexander Bartholomäus, Anke Becker, Michael Bitzer, Ezio Bonifacio, Peer Bork, Nicolas Casadei, Thomas Clavel, Maria Colome-Tatche, Andreas Diefenbach, Alexander Dilthey, Nicole Fischer, Konrad Förstner, Sören Franzenburg, Julia-Stefanie Frick, Gisela Gabernet, Julien Gagneur, Tina Ganzenmüller, Siri Göpel, Alexander Goesmann, Torsten Hain, André Heimbach, Michael Hummel, Angelika Iftner, Thomas Iftner, Stefan Janssen, Jörn Kalinowski, René Kallies, Birte Kehr, Andreas Keller, Sarah Kim-Hellmuth, Christoph Klein, Oliver Kohlbacher, Karl Köhrer, Jan Korbel, Denise Kühnert, Ingo Kurth, Markus Landthaler, Yang Li, Kerstin Ludwig, Oliwia Makarewicz, Manja Marz, Alice McHardy, Christian Mertes, Markus Nöthen, Peter Nürnberg, Uwe Ohler, Stephan Ossowski, Jörg Overmann, Klaus Pfeffer, Anna R. Poetsch, Alfred Pühler, Nikolaus Rajewsky, Markus Ralser, Olaf Rieß, Stephan Ripke, Ulisses Nunes da Rocha, Philip Rosenstiel, Antoine-Emmanuel Saliba, Leif Erik Sander, Birgit Sawitzki, Philipp Schiffer, Wulf Schneider, Eva-Christina Schulte, Joachim L. Schultze, Alexander Sczyrba, Yogesh Singh, Michael Sonnabend, Oliver Stegle, Jens Stoye, Fabian Theis, Janne Vehreschild, Jörg Vogel, Max von Kleist, Andreas Walker, Jörn Walter, Dagmar Wieczorek, Sylke Winkler, John Ziebuhr

**Affiliations:** 1 Systems Medicine, German Center for Neurodegenerative Diseases (DZNE), Bonn, Germany; 2 PRECISE Platform for Single Cell Genomics and Epigenomics at the German Center for Neurodegenerative Diseases and the University of Bonn, Bonn, Germany; 3 Genomics and Immunoregulation, Life & Medical Sciences (LIMES) Institute, University of Bonn, Bonn, Germany; 4 Department of Internal Medicine and Radboud Center for Infectious Diseases (RCI), Radboud University Medical Center, Nijmegen, The Netherlands; 5 4th Department of Internal Medicine, National and Kapodistrian University of Athens, Medical School, Athens, Greece; 6 Department I of Internal Medicine, University Hospital of Bonn (UKB), Bonn, Germany; 7 1st Department of Pulmonary Medicine and Intensive Care Unit, National and Kapodistrian University of Athens, Medical School, Athens, Greece; 8 West German Genome Center (WGGC), University of Bonn, Bonn, Germany; 9 Department of Intensive Care Medicine and Radboud Center for Infectious Diseases (RCI), Radboud University Medical Center, Nijmegen, The Netherlands; 10 Department of Gastroenterology, Hepatology and Infectious Diseases, University Hospital Düsseldorf, Heinrich Heine University Düsseldorf, Düsseldorf, Germany; 11 Population Health Sciences, German Center for Neurodegenerative Diseases (DZNE), Bonn, Germany; 12 Department of Internal Medicine II, Section of Pneumology, University Hospital of Bonn (UKB), Bonn, Germany; 13 Department of Neurology, Faculty of Medicine, University of Bonn, Bonn, Germany; 14 Immunology & Metabolism, Life and Medical Sciences (LIMES) Institute, University of Bonn, Bonn, Germany; 15 Institute for Medical Biometry, Informatics and Epidemiology (IMBIE), Faculty of Medicine, University of Bonn, Bonn, Germany; 16 German Center for Infection Research (DZIF), Bonn, Germany

**Keywords:** COVID-19, Blood transcriptomics, Transcriptome, Co-expression analysis, Stratification, Molecular disease phenotypes, Granulocytes, Neutrophils, Drug repurposing

## Abstract

**Background:**

The SARS-CoV-2 pandemic is currently leading to increasing numbers of COVID-19 patients all over the world. Clinical presentations range from asymptomatic, mild respiratory tract infection, to severe cases with acute respiratory distress syndrome, respiratory failure, and death. Reports on a dysregulated immune system in the severe cases call for a better characterization and understanding of the changes in the immune system.

**Methods:**

In order to dissect COVID-19-driven immune host responses, we performed RNA-seq of whole blood cell transcriptomes and granulocyte preparations from mild and severe COVID-19 patients and analyzed the data using a combination of conventional and data-driven co-expression analysis. Additionally, publicly available data was used to show the distinction from COVID-19 to other diseases. Reverse drug target prediction was used to identify known or novel drug candidates based on finding from data-driven findings.

**Results:**

Here, we profiled whole blood transcriptomes of 39 COVID-19 patients and 10 control donors enabling a data-driven stratification based on molecular phenotype. Neutrophil activation-associated signatures were prominently enriched in severe patient groups, which was corroborated in whole blood transcriptomes from an independent second cohort of 30 as well as in granulocyte samples from a third cohort of 16 COVID-19 patients (44 samples). Comparison of COVID-19 blood transcriptomes with those of a collection of over 3100 samples derived from 12 different viral infections, inflammatory diseases, and independent control samples revealed highly specific transcriptome signatures for COVID-19. Further, stratified transcriptomes predicted patient subgroup-specific drug candidates targeting the dysregulated systemic immune response of the host.

**Conclusions:**

Our study provides novel insights in the distinct molecular subgroups or phenotypes that are not simply explained by clinical parameters. We show that whole blood transcriptomes are extremely informative for COVID-19 since they capture granulocytes which are major drivers of disease severity.

**Supplementary Information:**

The online version contains supplementary material available at 10.1186/s13073-020-00823-5.

## Background

Pandemic spread of the recently emerged coronavirus, severe acute respiratory syndrome-coronavirus 2 (SARS-CoV-2), has resulted in over 84 million confirmed infected individuals and over 1.8 million deaths worldwide (WHO, covid19.who.int, as of January 6, 2021) from the resulting severe respiratory illness, called coronavirus disease 2019 (COVID-19) [1–3]. Based on clinical observations, it has become clear that there is great variety in disease manifestation, ranging from asymptomatic cases, to flu-like symptoms, to severe cases needing mechanical ventilation, to those who do not survive [4–8]. Increasing evidence suggests that the immune system plays a pivotal role in determining the severity of the disease course and it has been suggested that different molecular phenotypes might be responsible for the heterogeneous outcome of COVID-19 [9–14]. Identifying these molecular phenotypes might not only be important for a better understanding of the pathophysiology of the disease, but also to better define patient subgroups that are more likely to benefit from specific therapies [15–20]. Indeed, while vaccines are still under development, finding an effective and patient-tailored therapeutic management for COVID-19 patients including targeting derailed immune mechanisms [21–23] is key to mitigate the clinical burden as well as to prevent further disease fatalities [18, 19].

The analysis of peripheral blood-derived immune parameters in inflammatory and infectious diseases either by classical testing, including flow cytometry and serum protein measurements, or by omics technologies, including transcriptomics, has been proven very valuable in the past [24–32]. In COVID-19 patients, monitoring peripheral blood as a proxy for the ongoing changes within the circulating cells of the immune system has revealed lymphopenia to correlate with disease severity [33]. Single-cell analysis of blood-derived cells revealed downregulation of MHC molecules on monocytes and granulocytes [34], immune cell exhaustion [35], and a dysregulated myeloid cell compartment [34, 36] including dendritic cells [37] in a disease stage-dependent manner. Serial immune response analyses revealed four immune signatures represented by growth factors, two cytokine-defined phenotypes as well as a chemokine-defined phenotype [14]. While an early elevation in cytokine levels was associated with worse disease outcomes, patients with moderate COVID-19 displayed a progressive reduction in antiviral and antifungal immune responses [14]. Moreover, impaired type I interferon responses were seen in severe COVID-19 cases [38]. In another study, three distinct patient immunotypes were related to a poor clinical trajectory when combining flow cytometry, single-cell proteomics, and clinical observations [12]. Furthermore, several studies reported increased IL-6 serum levels to be a hallmark of COVID-19 [9, 13, 39–41], but also TNF and IL-8 [41]. A very recent large multi-omics longitudinal observational study identified a sharp transition between mild and moderate disease, indicating that targeting such a shift therapeutically might be beneficial for these patients [13].

Indeed, while one can envision mild and/or early cases to benefit from antiviral drug treatments currently under clinical investigation, more severe cases may benefit from treatment to mitigate the excessive systemic immune reactions resulting in progressing pneumonia and even respiratory failure associated with severe COVID-19 [4–9]. The detrimental role of the systemic inflammation in the late phase of the disease has become clear, as the elevated inflammatory signaling has been associated with disease morbidity [6, 9, 13, 38–42]. Thus, a better understanding of the dysregulation of the host response to the infection leading to immunopathology is urgently needed to dissect and comprehend the immune parameters accompanying the heterogeneous disease severity seen upon SARS-CoV-2 infection.

Based on previous experience with other infectious diseases [24–30], we hypothesized that whole blood transcriptomes should allow us to (1) determine immune cellular characteristics and functions in COVID-19 patients, (2) reveal heterogeneous molecular phenotypes of patients with similar clinical presentation, (3) define commonalities and differences of COVID-19 in comparison to other inflammatory conditions, and (4) predict potential drug repurposing that might counteract observed immune dysregulations.

Here, by using blood transcriptomes, we provide evidence for molecular subtypes within the immune response of COVID-19 patients beyond distinguishing mild and severe cases only. In addition, molecular changes in blood of severely affected patients are strikingly associated with changes in the granulocyte compartment. Furthermore, blood transcriptomes of molecular subtypes of COVID-19 patients seem to be unique in comparison to more than 2600 samples derived from other infections, inflammatory conditions, and controls. Finally, reverse drug target prediction using patients’ blood transcriptomes revealed known as well as additional new potential targets for further evaluation. Our data might also serve as a starting point for a large-scale assembly of molecular data collected during currently ongoing and future therapy trials for COVID-19 patients based on whole blood transcriptomes.

## Methods

### Human cohorts

#### Whole blood samples for RNA-seq analysis

The study was conducted between March 13 and March 30, 2020. A total of 6 ml of blood was sampled from patients with community-acquired pneumonia (CAP) by SARS-CoV-2 within the first 24 h of hospital admission. CAP was defined as the presence of diffuse infiltrates in chest X-ray or chest computed tomography and positive molecular testing of respiratory secretions for SARS-CoV-2. Exclusion criteria were infection by the human immunodeficiency virus, neutropenia, and any previous intake of immunosuppressive medication (corticosteroids, anti-cytokine biologicals, and biological response modifiers). The studies were conducted under the 23/12.08.2019 approval of the Ethics Committee of Sotiria Athens General Hospital and the 26.02.2019 approval of the Ethics Committee of ATTIKON University General Hospital. Written informed consent was provided by patients or by first-degree relatives in case of patients unable to consent. Patients were classified based on the WHO ordinal scale: mild = WHO1–4 and severe = WHO5–7. “Immune classification” of the patients is based on the criteria used in Giamarellos-Bourboulis et al. [40]: MAS for patients with > 4.420 ng/ml ferritin, dysregulation for patients with < 4.420 ng/ml ferritin with < 5000 molecules of HLA-DR+/CD14+ cells, and intermediate for those patients lying in between MAS and dysregulation. The following information was recorded: white blood cell count and differential, administered treatment, and 28-day outcome. Patients were sampled within 24 h upon admission to the hospital. A volume of 2.5 ml of the collected blood was transferred into one PAXgene tube and stored at − 80 °C. The remaining was used for flow cytometry analysis. A similar amount of blood was sampled from 10 controls, matched for age, sex, and Charlson’s comorbidity index. They were subject to testing of the nasopharyngeal secretion for SARS-CoV-2 and all confirmed to be asymptomatic and seronegative.

For the second cohort, whole blood samples were collected for RNA-seq analysis in PAXgene tubes from 30 patients upon admission to the Intensive Care Unit of the Radboud University Medical Center in Nijmegen, the Netherlands. The study was carried out in accordance with the applicable rules concerning the review of research ethics committees and informed consent. All patients or legal representatives were informed about the study details and could decline to participate. COVID-19 was diagnosed by a positive SARS-CoV-2 RT-PCR test in nasopharyngeal and throat swabs and/or by typical chest CT-scan findings. Exclusion criteria were hematological malignancies and/or active chemotherapy, solid organ transplant, autoimmune diseases, and pre-existent use of high-dose corticosteroids.

#### Granulocyte samples for RNA-seq analysis

This study was approved by the Institutional Review Board of the University Hospital Bonn (073/19 and 134/20). After providing written informed consent, 16 COVID-19 patients (44 samples) were included in the study. In-patients who were not able to consent at the time of study enrollment, consent was obtained after recovery. COVID-19 patients who tested positive for SARS-CoV-2 RNA in nasopharyngeal swabs were recruited at the Medical Clinic I of the University Hospital Bonn between March 30 and May 17, 2020. Longitudinal samples were included from day 1 to 20 after onset of symptoms and grouped into day 1–10 and 11–20 according to previous reports [34, 43].

Granulocytes were isolated from EDTA-treated or heparinized peripheral blood by density centrifugation over Pancoll or Ficoll-Paque density centrifugation (density: 1.077 g/ml). Granulocyte fractions were then treated with 10 ml RBC lysis buffer (Biolegend) for 10 min. After RBC lysis, cells were washed with DPBS and recovered by centrifugation at 300×*g* for 10 min. Granulocyte pellets were then lysed with 500 μl of QIAzol (Qiagen), shortly vortexed, and incubated 5 min at RT prior storage at − 80 °C until RNA extraction.

### Rhineland Study as control samples within the integrated dataset for disease comparison

#### Study population

The Rhineland Study is an ongoing community-based cohort study in which all inhabitants of two geographically defined areas in the city of Bonn, Germany, aged 30–100 years are being invited to participate. Persons living in these areas are predominantly German with Caucasian ethnicity. Participation in the study is possible by invitation only. The only exclusion criterion is insufficient German language skills to give informed consent.

#### Ethical approval

Approval to undertake the Rhineland Study was obtained from the ethics committee of the University of Bonn, Medical Faculty. The study is carried out in accordance with the recommendations of the International Conference on Harmonization (ICH) Good Clinical Practice (GCP) standards (ICH-GCP). Written informed consent was obtained from all participants in accordance with the Declaration of Helsinki.

#### Blood withdrawal

Overnight fasting blood was collected from all participants between 7:00 and 9:30 AM, including a PAXgene tube for RNA extraction.

### Flow cytometry techniques

Whole blood cells were incubated for 15 min in the dark with anti-CD45 PC5 (emission 667 nm, Beckman Coulter). Fluorospheres (Beckman Coulter) were used for the determination of absolute counts. Cells were analyzed after running through the CYTOMICS FC500 flow cytometer (Beckman Coulter Co, Miami, FL). Isotypic IgG controls stained also with anti-CD45 were used for each patient. Gating to identify neutrophils and lymphocytes was done by the characteristic sideward scattering of CD45-positive cells (Additional file 2: Figure S8).

### Whole blood RNA isolation

Total RNA was isolated from whole blood samples stored and stabilized in PAXgene RNA tubes using the Qiagen PAXgene Blood miRNA kit according to the manufacturer’s guidelines. Eluted RNA was dissolved in RNase-free water. The quality and quantity of RNA were evaluated by visualization of 28S and 18S band integrity on a Tapestation 4200 system (Agilent).

### RNA-sequencing

Total RNA was converted into double-stranded cDNA libraries using the TruSeq Stranded Total RNA with Ribo-Zero Globin kit (Illumina). In brief, ribosomal and globin mRNA were depleted from 750 ng purified total RNA using biotinylated, target-specific oligos combined with Ribo-Zero rRNA removal beads; remaining RNA was fragmented using divalent cations under elevated temperature. First-strand was generated using SuperScript2 RT (Invitrogen) supplemented with actinomycin D, followed by second-strand synthesis with dUTP replacing dTTP. 3′ ends were adenylated and index adapters were ligated before subsequent PCR amplification to yield the final library. Remaining overhangs were converted into blunt ends via exonuclease/polymerase activities, and enzymes were removed. Selective enrichment of DNA fragments with ligated adaptor molecules was performed using Illumina PCR primers in a 15-cycle PCR reaction, followed by purification cDNA using SPRIBeads (Beckman Coulter). Libraries were quantified by Qubit dsDNA HS Assay (Thermo Fisher Scientific), and fragment size distribution was determined using the HS D1000 assay on a Tapestation 4200 system (Agilent). High-throughput sequencing was carried out with a NovaSeq™ 6000 Sequencing System S2 (50bp paired-end reads), and data was converted into fastq files using bcl2fastq2 v2.20.

### RNA-sequencing analysis

Sequenced reads were aligned and quantified using STAR: ultrafast universal RNA-seq aligner (v2.7.3a) [44] and the human reference genome, GRCh38p13, from the Genome Reference Consortium. Raw counts were imported using DESeqDataSetFromHTSeqCount function from DESeq2 (v1.26.0) [45] and rlog transformed according to DESeq2 pipeline. DESeq2 was used for the calculation of normalized counts for each transcript using default parameters. All normalized transcripts with a maximum over all row mean lower than 10 were excluded resulting in 37,526 present transcripts. Differentially expressed genes were calculated for the scenario status (COVID-19 vs controls), mild/severe (severe COVID-19 vs controls, mild COVID-19 vs controls, and severe vs mild COVID-19), and new_cluster (1vs6, 2vs6, 3vs6, 4vs6, and 5vs6) separately using a *p* value cutoff of 0.05, an adjusted *p* value (IHW) < 0.05 (independent hypothesis weighting), and a FC of 2. All present transcripts were used as input for principal component analysis. The top 25% most variable transcripts within the dataset were selected and visualized in a heat map. DEGs were visualized as DE bar plots and were used as input for volcano plots.

### Gene ontology enrichment analysis (GOEA)

To test for functional enrichment within all three scenarios, we performed GOEA for up- or downregulated transcripts in the respective comparison using gene ontology set of biological processes. Gene set “c5.bp.v7.0.symbols.gmt” was obtained from the Molecular Signatures Database (MSigDB) [46]. compareCluster and enrichGo functions from the R package ClusterProfiler (v3.12.0) [47] were used to determine significant enrichment (*q* value < 0.05) of biological processes. All present genes were used as background (universe).

### Filtering for transcription factors, epigenome, surfaceome, and secretome

All present transcripts were filtered and sorted by their variance in the dataset. The 20 most variable genes of each category were selected and visualized using a heat map. Transcription factor lists were extracted from [48], the epigenome gene list was literature-driven, and surface and secretome markers were extracted from the Human Protein Atlas [49].

### Clustering of patients according to clinical parameters

The contribution of each clinical parameter to the transcriptome in COVID-19 patients was determined using linear modeling of each parameter separately with PC1. Clinical parameters with rounded up adjusted *r*-square ≥ 0.2 were used for agglomerative hierarchical clustering of the COVID-19 patients. A dissimilarity matrix based on Gower distance was calculated using the daisy function from the cluster packages (version 2.1.0). Agglomerative hierarchical clustering was performed using the hclust function, defining the method with a setting forward.D2 method linkage. We evaluated the clustering by extracting cluster statistics using the function cluster.stats from the package fpc (version 2.2-5). The number of clusters was chosen at the value at which the lowest distance among patients within clusters (i.e., low value of within-cluster sum of squares distance) and preserving a high distance among clusters (i.e., high average silhouette width) was achieved, while still maintaining a comparable number of individuals among the clusters.

### Linear support vector regression

Linear support vector regression [50] was employed to computationally deconvolute the study’s whole blood samples. Gene expression tables were normalized with DESeq2 and were utilized as the input mixture file. LM22-subsetted signatures for B cells, T cells, NK cells, monocytes, dendritic cells, eosinophils, and neutrophils were generated as described on https://cibersort.stanford.edu/tutorial.php. The algorithm was subsequently run with 1000 permutations, and the proportions of cell types were visualized with ggplot2 (v3.2.1) [51].

### CoCena^2^: Construction of Co-expression network analysis—automated

To define differences and similarities in transcript expression patterns among the different groups, CoCena^2^ (Construction of Co-expression network analysis—automated) was performed based on Pearson’s correlation. CoCena^2^ is a network-based approach to identify clusters of genes that are co-expressed in a series of observed conditions based on data retrieved from RNA-sequencing. The tool offers a variety of functions that allow subsequent in-depth analysis of the biological context associated with the found clusters. First, we have calculated the variance for each gene in the complete dataset. Nine thousand three hundred seventy-eight of all present genes show a variance of at least 3rd quantile of all variances. Therefore, we selected the 10,000 most variable genes as input for the analysis.

To identify genes whose expression patterns are highly similar across all tested samples, pairwise Pearson’s correlation coefficients are calculated using the R package Hmisc (v4.1-1). The underlying assumption of the Pearson correlation to the data is that it is normally distributed, which is a valid assumption to make in the context of gene expression when looking at expression patterns within different experimental conditions. The correlation between each pair of genes is the basis for the subsequent network construction. Therefore, the tool focuses mainly on positively correlated gene pairs, since the rate of confirmation of an edge representing an association of genes is higher than that of a non-existing association.

In order to refine the structure of the upcoming network and to unravel the condition-specific signatures, a correlation cutoff is proposed to mark the minimal correlation a pair of genes must exhibit for their co-expression to be taken into account. The cutoff is determined based on different criteria:

1. Scale-free topology

Gene expression networks have been argued to have a scale-free topology [52], meaning that the majority of vertices has a low number of adjacent edges, also referred to as the vertex’ degree, whereas only very few vertices have a high degree. The degree distribution of scale-free networks asymptotically follows a power law. To assess the scale-free topology of a network constructed by a given correlation cutoff, a log-log plot of the degree distribution is constructed and the *R*^2^ value of the resulting linear regression is used to evaluate the scale-free criterion.

2) Number of graph components

A graph component is a subset of nodes, such that there is a path from every node within the component to any other node in that same component but none connecting the nodes to any outside of that component. Even though there exist functional collections of genes that cooperate to fulfill a common task, these collections are not expected to be operating independently within the cell. Thus, the cutoff proposal favors graphs with a small number of components.

3) Number of edges

To avoid a highly connected graph with great lack of structure—“hairball,” the cutoff is chosen such that the number of edges is minimized while respecting the abovementioned criteria.

A Pearson correlation coefficient cutoff of 0.857 (6085 nodes and 252,584 edges) was chosen to construct scale-free networks.

The undirected co-expression network is constructed based on the gene pairs which show a higher correlation in their expression pattern than the set cutoff. A series of network-based clustering algorithms is available to then identify clusters of strong co-expression within the network. An option “auto” is provided, which tests the different clustering algorithms and picks the one that achieves the highest modularity score. Unbiased clustering was performed using the “label propagation” algorithm in igraph (v1.2.1) [the igraph software package for complex network research] and was repeated 1000 times. Modules with less than 40 genes were discarded. Genes assigned to more than 5 different clusters during the iterations received no cluster assignment.

To assess the expression strength of the found gene clusters in the different studied conditions, the group fold changes (GFCs) of the conditions are calculated for each gene by calculating the mean expression of a gene over all samples and then computing the fold change of the mean gene expression within each condition from the overall mean. The GFCs of all genes within one cluster are then added and divided by the total number of genes per cluster, resulting in condition-specific GFCs per cluster. By using the GFC, it is possible to illustrate a directional change for all conditions including the control samples in respect to the overall GFC. Agglomerative hierarchical clustering was performed by the hclust function (cluster package, version 2.1.0), using a dissimilarity matrix of samples based on the GFC values of each sample defined with the daisy function for calculating the Euclidean distances. The number of clusters was set to achieve a low within-cluster sum of squares distance and a high average silhouette, while preserving a comparable number of individuals within each cluster. The clinical parameters and the GFC results are displayed in a heat map where conditions are clustered by their GFCs revealing similar and opposing patterns (Cluster/Condition heat map). The expression pattern of the modules can be further used for additional analysis, e.g., stratification in another cohort.

Utilizing the R package *clusterProfiler*, CoCena^2^ automatically analyzes the gene clusters with respect to different kinds of gene set enrichments: the genes within each cluster are scanned for enrichment in KEGG [53], Hallmark [54], Gene Ontology terms [55], and Reactome [56]. Using the R package *pcaGoPromoter* [57], the genes are also analyzed for enrichment of transcription factor binding sides, and if the predicted transcription factors are present in the data, their expression profile is visualized to facilitate evaluation of their possible role.

To investigate the interactions between protein-coding and long non-coding RNAs, we utilized the enricher function from the clusterProfiler package. We performed an enrichment analysis for lncRNA species, using the protein-coding genes that belong to the lightgreen cluster as the input gene list and all the network protein-coding genes as background. The annotation table defining lncRNA to protein-coding RNA was downloaded from the RNA interactome database RNAInter [58], filtered to only include interactions of lncRNA detected by the RNA-sequencing, had an experimental validation score of at least 0.5, and was involved in regulating the function of granulocytes [59]. Next, to obtain a comprehensive understanding of the lncRNA that may be relevant for this specific network module, the lncRNA found by the enrichment analysis with *p* value < 0.1 were sorted according to the highest number of genes. Thereafter, Spearman’s correlation among the gene expression of each lncRNA and its corresponding protein-coding RNAs was performed, and significant protein-coding RNA genes were plotted in a heat map. The CoCena^2^ network was visualized by using the ggplot function from the ggplot2 package. Annotations were generated by filtering the edges of the network for the 5 top connected transcription factors, epigenetic regulators, and surface and secretome markers in each cluster. GO enrichment analysis was performed on each cluster by utilizing the enrichGO function from the clusterProfiler package to assess the overall functionality of the cluster using the genes of each cluster as the input and all the in the network as background. The top GO term and top connected genes of each cluster were compiled representing their general characteristic.

### Granulocyte dataset analysis

Granulocyte raw data was aligned and quantified using STAR (v2.7.3a) and the human reference genome, GRCh38p13, from the Genome Reference Consortium. Raw counts were imported using DESeqDataSetFromHTSeqCount function and rlog transformed. DESeq2 was used for the calculation of normalized counts for each transcript using default parameters. All normalized transcripts with a maximum over all row mean lower than 10 were excluded resulting in 27,781 present transcripts. Differentially expressed genes were calculated for the severe vs mild for day 1–10 and 11–20 (post 1st symptoms groups) separately using a *p* value cutoff of 0.05, an adjusted *p* value (IHW) < 0.05 (independent hypothesis weighting), and a FC of 2. All present transcripts were used as input for principal component analysis. DEGs were visualized as DE bar plots.

To visualize the module expression over the time between mild and severe case, the granulocyte data was grouped by the modules identified in Fig. 2 and the function geom_smooth with default parameters was used to calculate the estimated curve for the module gene expression over the time and a confidence band representing the uncertainty in the estimate.

### Data integration for disease comparison

To describe the differences and similarities between COVID-19 and other diseases, we searched in databases for genomics data such as Gene Expression Omnibus (GEO) [60] and ArrayExpress [61] for studies that fulfill certain criteria: (I) having at least 20 samples, (II) the disease of study was of relevance (other infections, such as bacterial and viral, plus diseases that mainly involve immune dysregulation, such as autoimmune disease), and (III) library preparation and sequencing technology differ as little as possible from our COVID-19 protocol, except for the influenza dataset which comes from a microarray experiment (GSE111368). The fastq files of 18 additional studies (PRJNA588242, GSE101705, GSE107104, GSE112087, GSE127792, GSE128078, GSE129882, GSE133378, GSE143507, GSE57253, GSE63042, GSE66573, GSE79362, GSE84076, GSE89403, GSE90081, GSE97590, GSE99992, and the Rhineland study) were downloaded and aligned with STAR. The counts were imported into R (v3.6.2) and were modeled for each gene using DESeq2. Merged raw counts from the RNA-seq studies were combined with the microarray study and were filtered for the genes present in the COVID-19 co-expression network, and ribosomal protein-coding genes and mitochondrial genes were removed, yielding a total of 5770 genes and 3176 samples. To account for differences in sequencing depth across studies as well as between RNA-seq and microarray data, a quantile normalization was performed on the filtered data. Group fold changes were calculated, where the grouping variable was set to be the disease status.

To explore COVID-19 associated expression of genes within the integrated dataset, the data was intersected with the gene modules previously retrieved from the COVID-19 CoCena^2^ network, and the mean group fold changes were determined per cluster and condition and visualized in a heat map.

The modules were analyzed for enriched immune cell markers as provided by CIBERSORT and BD Rhapsody, and those that showed neutrophil enrichment were screened for genes representative of different neutrophil subtypes as recently described [34].

### Enrichment of signature from scRNA data of granulocytes

The signatures of different neutrophil states in COVID-19 as previously described [34] were enriched for the different clusters from CoCena^2^.

To get a more fine-grained differentiation of the specific neutrophil states for Fig. 3, the authors kindly provided additional signatures from the scRNA dataset using a Wilcoxon rank sum test for differential gene expression implemented in Seurat. Genes had to be expressed in > 10% of the cells of a cluster, to exceed a logarithmic threshold > 0.1, and to have > 5% difference in the minimum detection between two clusters. The following additional comparisons were performed: 8 and 9 (pre- and immature neutrophils combined) vs the rest, and 1, 3, 4, and 6 (neutrophil states from control patients) vs the rest. To get unique signature genes for clusters 0, 2, and 5 (COVID-19-specific clusters), we took the following approach for each cluster: (1) calculate DEG for cluster 0 vs all other clusters, (2) calculate DEG for cluster 0 vs 2 and 5, (3) take intersection of these two calculations, and (4) remove genes that occur in more than one of these intersections of cluster 0, 2, or 5.

### Gene set enrichment analysis (GSVA)

The GSVA R package (v1.34.0) [62] was used to test the enrichment of neutrophil signatures [34] in the normalized gene expression table. The *gsva* method was used for the run and data were visualized in a heat map with the pheatmap (v1.0.12) package.

### Overview of drugs

An overview of currently used, recommended, or investigated drugs for treatment of COVID-19 patients was compiled from drug lists and lists of drugs in clinical trials downloaded from https://www.drugbank.ca/covid-19, https://www.pharmgkb.org/page/COVID, and https://clinicaltrials.gov/ct2/results?cond=COVID-19 (last update: 5 June 2020). Classification of the drugs was performed based on the ATC code, as well as additional research on the drugs action. Drug target genes were identified using the DrugBank database [63] (Additional file 7: Table S6). The number of drugs currently recommended or investigated, and the number of clinical trials within the respective drug classes were visualized using the ggplot2 package [64, 65]. The target genes of the drugs currently recommended or investigated with a minimum frequency of 4 were visualized in a word cloud using the wordcloud package (version 2.6).

### Drug prediction

To identify drugs, which reverse the gene expression signature observed in the comparisons of the COVID-19-specific clusters compared to the control cluster, the drug prediction databases iLINCS (http://www.ilincs.org/ilincs/) and CLUE (https://clue.io/) were accessed. As input for the drug prediction, the top 1000 (iLINCS) or the top 100 (CLUE) DEGs were used. Drugs reversing the COVID-19 gene expression signature (defined by a negative score) were pooled together with drugs under investigation in current literature, resulting in a list of 940 unique drugs. Using the iLINCS API (https://github.com/uc-bd2k/ilincsAPI/blob/master/usingIlincsApis.Rmd), every gene expression signature from each drug listed in the signature libraries iLINCS chemical perturbagens (LINCSCP), iLINCS targeted proteomics signatures (LINCSTP), Disease-related signatures (GDS), Connectivity Map signatures (CMAP), DrugMatrix signatures (DM), Transcriptional signatures from EBI Expression Atlas (EBI), Cancer therapeutics response signatures (CTRS), and Pharmacogenomics transcriptional signatures (PG) was downloaded. Labeling was performed in the following principle: “drug name”_“database”_“database ID”. Signatures were ordered by fold change, and only the top 300 genes were used. This resulted in a total of 62,897 unique drug signatures each with an up- and downregulated set. Subsequently, GSEA [66] was performed on the sequencing data for every up- and downregulated set for each drug and each cluster comparison. The resulting normalized enrichment scores (NES) were used to calculate the delta NES for each drug, defined as ΔNES = NES (down) − NES (up), ergo the difference of the NES from the downregulated set and the NES from the upregulated set of each respective drug. These ΔNES values were then *k*-mean clustered (*k* = 40). The clusters showing the highest ΔNES values for all comparisons and the cluster showing only high ΔNES in the comparison G1 vs G6 (most severe) were chosen and selected ones of the uniquely present drugs shown. The leading edge genes of the downregulation signatures of these drugs for the G1 vs G6 comparison were examined, and the frequency was counted. Recurring target genes were plotted on the CoCena^2^ network.

Patterns of differential gene expression of genes targeted by drugs which are currently approved or under investigation for the treatment of COVID-19 patients were visualized using ggplot2. To this end, target genes of each drug and their first-degree neighbors were extracted from several databases and the gene co-expression networks, respectively. Regulation patterns of expression of these genes in different COVID-19 patient groups, as compared to the control group, were classified as up-/downregulated or not significant (n.s.) when pairwise comparisons of gene expression of COVID-19 patients and controls were not statistically significant. The same methodology was applied to genes not included in the drug-target list to identify genes which are not targeted by current drugs but could be potentially targeted by newly identified drugs.

## Results

### Whole blood transcriptomes reveal diversity of COVID-19 patients not explained by disease severity

**Fig. 1:**
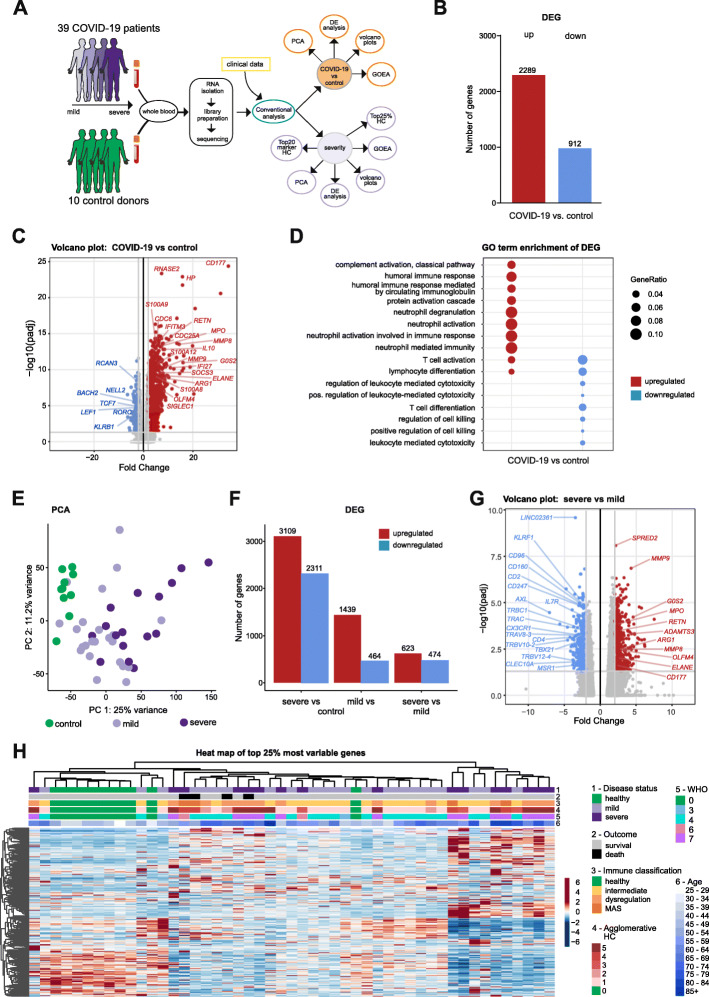
Whole blood transcriptomes reveal diversity of COVID-19 patients not explained by disease severity. **a** Schematic workflow for analysis of whole blood transcriptome data. **b** Number of significantly upregulated (red) and downregulated (blue) genes (FC > |2|, FDR-adj. *p* value < 0.05) comparing COVID-19 and control samples. **c** Volcano plot depicting fold changes (FC) and FDR-adjusted *p* values comparing COVID-19 and control samples. Differentially expressed up- (red) and downregulated genes (blue) are shown and selected genes are highlighted. **d** Plot of top 10 most enriched GO terms for significantly up- and downregulated genes, showing ratio of significantly regulated genes within enriched GO terms (GeneRatio). **e** PCA plot depicting relationship of all samples based on dynamic gene expression of all genes comparing mild and severe COVID-19 as well as control samples. **f** Number of significantly upregulated (red) and downregulated (blue) genes (FC > |2|, FDR-adj. *p* value < 0.05) comparing mild and severe COVID-19 as well as control samples. **g** Volcano plot depicting fold changes and FDR-adjusted *p* values comparing mild and severe COVID-19 as well as control samples. Differentially expressed up- (red) and downregulated genes (blue) are shown and selected genes are highlighted. **h** Hierarchical clustering map of 25% most variable genes between control patients and COVID-19 mild or severe patients, with additional annotation of disease outcome, hierarchical agglomerative clustering of clinical parameters COVID-19, the groups defined by agglomerative clustering, WHO ordinal score, and age bins

To investigate the host immune response of COVID-19 patients in a systematic approach, whole blood transcriptomes were analyzed from 39 patients and 10 control donors recruited at the same hospital by RNA-sequencing (RNA-seq, Fig. 1a, Additional file 1: Table S1). Two-dimensional data representation using principal component analysis (PCA) showed separation of COVID-19 and control samples (Additional file 2: Figure S1A). Differential expression analysis identified 2289 upregulated and 912 downregulated genes comparing COVID-19 and control samples (FC > |2|, padj < 0.05; Fig. 1b). Upregulated genes showed greater fold changes than the downregulated genes (Fig. 1c). Of note, *CD177*, markedly expressed in neutrophils [67, 68], was the most prominently upregulated gene with the lowest *p* value. Heightened expression was further found for several granulocyte- and monocyte-associated molecules, such as Eosinophil-derived neurotoxin (*RNASE2*), Haptoglobin (*HP*), Neutrophil elastase (*ELANE*), Olfactomedin 4 (*OLFM4*), Myeloperoxidase (*MPO*), Resistin (*RETN*), matrix metalloproteinases (*MMP8*, *MMP9*), and alarmins (*S100A8*, *S100A9*, *S100A12*), as well as for cell cycle progression-associated genes (*G0S2*, *CDC6*, *CDC25A*), type I interferon (IFN)-induced genes (*IFI27*, *IFITM3*, *SIGLEC1* (CD169)), but also genes with immunosuppressive functions (*IL10*, *SOCS3*, *ARG1* (Arginase)). Downregulated genes included many lymphocyte-associated factors, such as *NELL2*, *RORC*, *KLRB1*, *TCF7* (TCF1), *RCAN3* (Calcipressin-3), *BACH2*, or *LEF1* (Fig. 1c, Additional file 3: Table S2). Functional analysis of the differentially expressed genes (DEGs) by gene ontology enrichment analysis (GOEA) revealed granulocyte and complement activation-associated terms enriched in the upregulated DEGs and lymphocyte differentiation and T cell activation for the downregulated DEGs (Fig. 1d). Interestingly, the T cell activation-associated genes accounting for the enrichment of this term for the upregulated DEGs included *IL10* and *CD274* (PD-L1) pointing at suppressive T cell functionality (Additional file 3: Table S2).

Given the heterogeneous nature of clinical manifestation of COVID-19, we sought to stratify the transcriptomic profiles by disease severity based on WHO ordinal scale. Classification scores of 1–4 was considered as “mild” and 5–7 as “severe.” Indeed, samples from patients with mild disease clustered more closely to the control samples, while those of severe cases scattered away in the PCA (Fig. 1e). Consequently, there was a greater number of DEGs in blood samples from severe COVID-19 patients than in mild patients when compared to controls (Fig. 1f). Many of the DEGs found in the COVID-19 vs control comparison (Fig. 1c) were also found when separating the COVID-19 samples by severity (Additional file 2: Figure S1B,C). Both, severe and mild COVID-19 in comparison to controls shared neutrophil-specific *CD177* and *HP* expression among the most upregulated DEGs, as well as lymphocyte-associated genes such as *ABLIM1*, *NELL2*, *RCAN3*, *RORC*, *BACH2*, and *KLRB1*, among the downregulated genes (Additional file 2: Figure S1B,C). GOEA reflected these findings (Additional file 2: Figure S1D). Although all samples from COVID-19 patients showed functional enrichment for granulocyte/neutrophil activation-associated terms in general, direct comparison of severe and mild COVID-19 patients revealed this to be a heightened characteristic of the immunoprofiles in severe COVID-19 (Additional file 2: Figure S1D). Upregulated DEGs in the severe vs mild sample comparison included *CD177*, Neutrophil elastase (*ELANE*), Olfactomedin 4 (*OLFM4*), Myeloperoxidase (*MPO*), Resistin (*RETN*), and matrix metalloproteinases *MMP8* and *MMP9*. Whereas the type I IFN-response genes, such as *IFI27* or *IFITM3*, were not differentially regulated in severe vs mild samples, expression of immunosuppression-associated factor Arginase (*ARG1*) was more pronounced in severe COVID-19 patients (Fig. 1g, Additional file 3: Table S2). Moreover, blood transcriptomes from severe cases showed decreased expression of lymphocyte-associated genes, such as the T cell receptor chains (*TRAC*, *TRBC1*), CD3 zeta chain (*CD247*), *CD4*, *CD2*, *TBX21* (TBET), and *IL7R*, as well as monocyte-associated genes, such as the fractalkine receptor (*CX3CR1*) or the macrophage scavenger receptor (*MSR1*) (Fig. 1g, Additional file 3: Table S2). Differences in gene expression were not restricted to granulocyte and T cell functions only: assessing the changes in defined gene groups, e.g., transcription factors, epigenetic regulators, and surface or secreted molecules, we observed many significant changes in genes that are not restricted to granulocytes or T cells, clearly indicating that other cell types are also transcriptionally altered in COVID-19 patients (Additional file 2: Figure S1E).

Distribution of the COVID-19 samples in the PCA revealed heterogeneity in the transcriptomic profiles (Fig. 1e), which might be due to clinical heterogeneity (Additional file 1: Table S1). In order to investigate this further, the top 25% of the most variable expressed genes were visualized in a heat map and samples sorted by unbiased hierarchical clustering based on their transcriptomic profiles, which resulted in more than three clusters suggesting higher transcriptional heterogeneity as explained by mild and severe COVID-19 cases vs control (Fig. 1h). Strikingly, neither disease, disease severity, nor the inclusion of outcome or immune classification [40] sufficiently explained the structure in the data. In order to get a better clinical understanding of the transcriptional data, we included further clinical parameters and grouped the COVID-19 patients accordingly (Fig. 1h). We therefore performed agglomerative hierarchical clustering using the clinical parameters that contributed most to the transcriptional differences observed across the first principal component of the dataset (*r*-adjusted square ≥ 0.1, Additional file 2: Figure S1F). The COVID-19 patients were clustered into five clinical groups, which was the optimal number of clusters at which the intra-group variance was low and the “clusters distance” remained high (Additional file 2: Figure S1G,H). Interestingly, neither COVID-19 disease status, immune classification, nor our clinical parameter-based grouping of the COVID-19 patients aligned with overall transcriptional variability in the data (Fig. 1h), indicating that hidden information in the blood transcriptome may guide further patient stratification.

### Co-expression analysis discloses COVID-19 subgroups with distinct molecular signatures

**Fig. 2:**
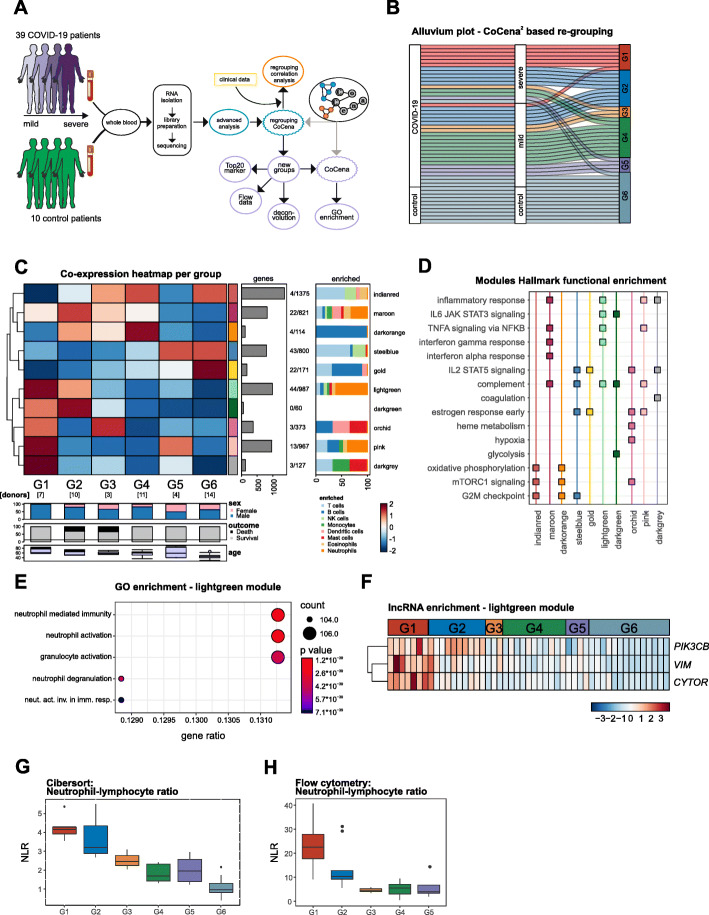
Co-expression analysis discloses COVID-19 subgroups with distinct molecular signatures. **a** Schematic overview of the analysis performed on the whole blood samples. **b** Alluvium plot visualizing the distribution of the samples according to different grouping; disease status, severity, and data-driven sample groups. **c** Group fold change heat map and hierarchical clustering for the six data-driven sample groups and the gene modules identified byCoCena^2^ analysis. **d** Functional enrichment of CoCena^2^-derived modules using the Hallmark gene set database. Selected top terms were visualized. **e** Functional enrichment of CoCena^2^ module lightgreen using GO gene set database. Top 5 terms were visualized. **f** Heat map presenting the normalized expression values of the lncRNA CYTOR, and protein-coding RNAs PIK3CB and VIM from the lightgreen CoCena^2^ module. **g** Neutrophil-lymphocyte ratio plot after cell type deconvolution at lineage level. **h** Neutrophil-lymphocyte ratio across the six data-driven sample groups. Box plots show median with variance, with lower and upper hinges representing the 25th and 75th percentile, respectively

Classical approaches to analyze the transcriptome data by using differential gene expression analysis based on sample groups defined by a selection of clinical parameters precluded dissection of the heterogeneity of the host immune response towards SARS-CoV-2 infection, which is evident in the high-parameter space of the transcriptome (Fig. 1). Co-expression analysis on the other hand identifies similarly regulated genes across samples and groups these genes into modules, which can then be explored for each patient sample individually or for entire patient groups. Applying such an approach using our established CoCena^2^ pipeline [https://github.com/Ulas-lab/CoCena2] (Fig. 2a) for all 49 samples (39 COVID-19, 10 control) independent of their clinical annotation disclosed 10 co-expression modules, designated by color indianred to darkgrey, across a total of 6085 genes included in the analysis (Additional file 2: Figure S2A). Hierarchical clustering of the samples based on their group fold changes (GFCs) for each module revealed a data-driven patient stratification assorting the samples into six groups (Additional file 2: Figure S2B), which were subsequently used in all following analyses: five different COVID-19 sample-containing groups, which only partially grouped by disease severity and illustrated heterogeneity of the immune response in COVID-19 patients, plus one group containing all control as well as four COVID-19 samples (Fig. 2b + Additional file 2: Figure S2C). Overlaying this information onto the original PCA reflected structured sample stratification as the newly defined groups clustered together (Additional file 2: Figure S2D). GFC analysis of the newly generated groups revealed group-specific enrichment of co-expressed gene modules (Fig. 2c). GOEA on each of the modules identified associated gene signatures displaying distinct functional characteristics, which distinguish the different sample groups G1–G6 (Fig. 2d + Additional file 2: Figure S3, Additional file 4: Table S3). For example, “inflammatory response” was enriched in modules maroon, lightgreen, pink, and darkgrey, all characteristic for sample groups G1 and G2 to different extents, indicating these to possibly undergoing a more vigorous inflammatory immune reaction (Fig. 2c, d). Of note, G1 and G2 harbor a great fraction of samples from patients with severe COVID-19 (Fig. 2b). Only a slight increase in the inflammation-associated module maroon, an increase in expression in the genes of darkorange (enriched in oxidative phosphorylation, mTORC1 signaling, and cell cycle-associated genes), and a loss of expression in the gold module (connected to estrogen response genes and IL2 signaling) were indicative of the G4 sample group. G6, encompassing all control samples, was not associated with any modules connected to inflammatory processes, but showed higher expression of indianred, steelblue, and gold, all functionally enriched basic cellular and metabolic processes. Extended analysis of the lightgreen module, containing 987 genes, revealed a prominent enrichment of granulocyte/neutrophil activation-related signatures (Fig. 2e, Additional file 4: Table S3). To further explore this neutrophil activation signature association, we investigated possible co-expression patterns of long non-coding RNAs (lncRNA) that were reported as regulators of granulocyte function [59]. *CYTOR* (also known as *Morrbid*) is a lncRNA that mediates survival of neutrophils, eosinophils, and classical monocytes in response to pro-survival cytokines [59], and interacts with the protein-coding RNAs for the catalytic PI3K isoform phosphatidylinositol-4,5-bisphosphate 3-kinase catalytic subunit beta (*PIK3CB*) and the filament Vimentin (*VIM*) [69]. Interestingly, expression of *CYTOR* was significantly increased in severe COVID-19 patient group G1 (*p* < 0.001) and correlated with both *PIK3CB* (*r* = 0.53, *p* < 0.001) and *VIM* (*r* = 0.55, *p* < 0.001) (Fig. 2f).

Next, we asked whether the enrichment for neutrophil activation-associated signatures in G1 and G2 is attributed to an increased relative number of granulocytes within the whole blood sample. Deconvolution of the expression values using linear support vector regression [50] showed increased relative percentages of neutrophils especially in G1 and G2 (Additional file 2: Figure S2E). G5, on the other hand, clearly displayed an increased percentage of monocytes. At the same time, lymphocyte enrichment was found to be reduced in the COVID-19 sample groups, most prominently in G1 and G2 (Additional file 2: Figure S2E). The linear deconvolution results were then validated by flow cytometry. Blood composition of COVID-19 donors confirmed an increased number of neutrophils and a decreased number of lymphocytes especially in G1 and G2 (Additional file 2: Figure S2F). As a result, the neutrophil-lymphocyte ratio (NLR), a clinical marker proposed for disease severity as it has been associated with an increased systemic inflammation [70, 71], was markedly elevated in G1 and G2 compared to the control sample-containing G6, both in the computationally deconvoluted results (Fig. 2g) as well as measured by flow cytometry (Fig. 2h). Interestingly, in context of the observation that men more often progress to severe COVID-19 than women [72], G1 encompasses samples from solely male patients (Additional file 2: Figure S2C). Analysis of the top 20 differentially expressed transcription factors, epigenetic regulators, and surface or secreted proteins for the six sample groups confirmed an increased inflammatory state, again most remarkably for G1 and G2, evident from the transcription factors of the STAT family, *STAT1*, *STAT3*, *STAT5B*, and *STAT6*; the surface marker *CSF3R* (G-CSF) or *FCGR3B* (CD16b); the secreted factors *GRN* or *IL1B*; or the epigenetic regulator *PADI4* (PAD4) (Additional file 2: Figure S2H).

We confirmed our findings of distinct molecular phenotypes in the blood of COVID-19 patients in a second independent cohort. Thirty patients, severely affected by SARS-CoV-2 infection, were sampled upon admission to the ICU. We stratified the obtained blood transcriptomes based on the module signatures from the co-expression analysis (Fig. 2c). The samples of the second cohort were filtered for the genes present in the COVID-19 co-expression network, group fold changes were calculated across all patients individually, and sample groups G1–G6 assigned according to their combinatorial module expression (Additional file 2: Figure S4A). Controls from the first cohort were included for comparison. Interestingly, in these ICU patients, we noted the transcriptome profiles from the second cohort to show greatest similarity to G1 and G2, which is in line with their severe phenotypes and our findings from the first cohort. Hierarchical clustering of the samples based on their group fold changes for each module stratified the samples of the second cohort into four groups (Additional file 2: Figure S4B). The control samples from the first cohort built one separate group, which we designated again as G6. To allow for group-specific comparison to the stratification within the first cohort (Fig. 2c), we calculated the mean GFCs of the four groups identified in the second cohort (Additional file 2: Figure S4C). Second cohort samples of the first group showed enrichment in modules lightgreen, pink, and darkgrey and were thus assigned most similar to G1; the third group of the new samples showed enrichment in modules maroon and darkorange, most similar to G2; and the remaining samples were stratified into an intermediate group exhibiting stronger expression of genes from the darkorange as well as pink module indicating characteristics of both G1 and G2 (Additional file 2: Figure S4C).

Collectively, co-expression analysis (CoCena^2^) in whole blood transcriptomes reveals at least five molecular phenotypes of the host’s immune response in COVID-19 patients with at least two different groups in clinically described severe COVID-19 patients. The two molecularly defined groups G1 and G2 are transcriptionally characterized by a pronounced neutrophilic signature, at the same time distinct in other cellular characteristics. Such molecular classification might serve as a basis for identifying clinical surrogates for patient stratification. Since whole blood transcriptomics captures functional changes in the host’s peripheral immune response across all cell types, we next sought a more detailed investigation of the granulocyte compartment within the framework of the newly identified subgroups.

### Granulocytes from severe COVID-19 patients show a simultaneous increase in inflammatory and suppressive signatures

**Fig. 3:**
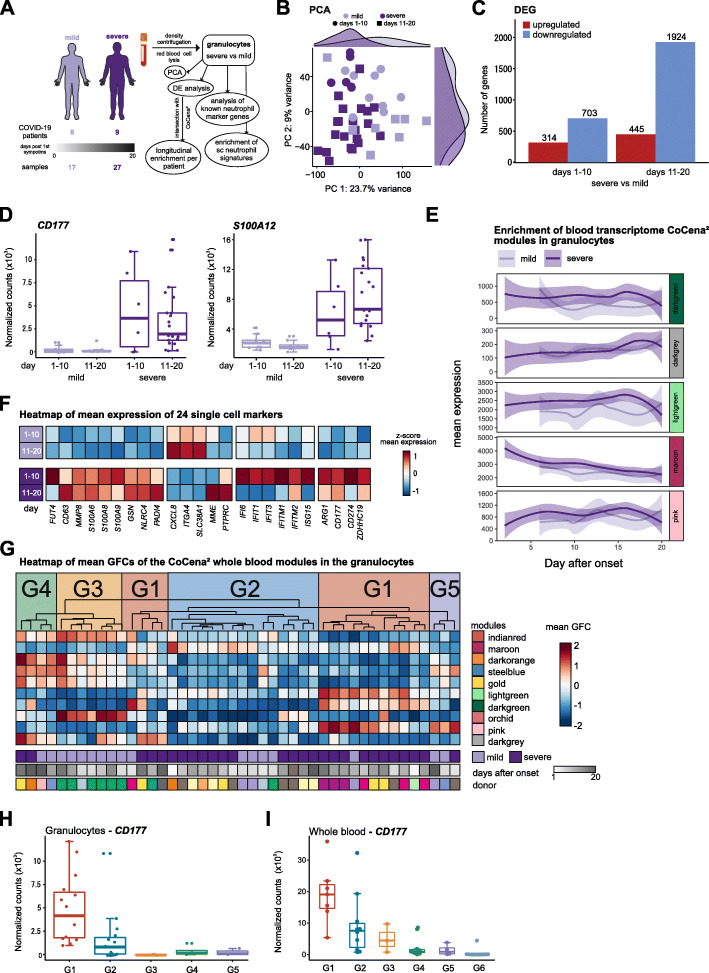
Granulocytes from severe COVID-19 patients show a simultaneous increase in inflammatory and suppressive signatures. **a** Schema of sample processing and analysis. **b** PCA of all genes within the dataset mapped by COVID-19 severity status. **c** Bar plot of DEGs between severe and mild COVID-19 patients at day 1–10 (left) and day 11–20 (right) (FC > |2|, FDR-adj. *p* value < 0.05). **d** Boxplot of CD177 (left) and S100A12 (right) in mild and severe COVID-19 patients at day 1–10 and 11–20. **e** Mean of group fold changes (GFCs) of the modules darkgreen, darkgrey, lightgreen, maroon, and pink in the granulocyte samples of mild (light purple) and severe (purple) COVID-19 cases over time. **f** Heat map of mean expression of 24 markers in mild (top) and severe (bottom) patients ordered by days after disease onset bins (day 1–10 and 11–20). **g** Heat map of mean GFCs of the CoCena^2^ whole blood modules in the granulocyte samples from each individual patient. Patients are clusters by the mean GFC module expression. Severity patterns found in the whole blood CoCena^2^ network were identified and patient groups were assigned accordingly (G1–G5). **h** Box plot of CD177 expression in granulocytes grouped by G1–G5. **i** Box plot of CD177 expression in whole blood grouped by G1–G6

To investigate whether the activation signatures seen in whole blood of COVID-19 patients are not only due to disease-associated increase of the neutrophil population, granulocytes were sequenced and transcriptomes were analyzed from 16 longitudinally sampled patients (8 mild, 9 severe), resulting in 17 mild and 27 severe COVID-19 samples (Fig. 3a). Evaluation of the relative cell type composition within each sample using linear deconvolution predicted the samples to mainly consist of neutrophils, with comparable fractions of 79% on average (Additional file 2: Figure S5A). Exploratory analysis by PCA showed a separation between mild and severe COVID-19 patients’ granulocyte samples, especially for the day 1–10 groups (Fig. 3b). Differential expression analysis identified 314 upregulated and 703 downregulated genes comparing severe and mild samples from day 1 to 10 after first symptoms, while comparison at a late disease stage showed less differences on gene level (445 up- and 1924 downregulated genes; FC > |2|, padj < 0.05; Fig. 3c, Additional file 5: Table S4). Whole blood transcriptome analysis showed enrichment of neutrophil activation-associated signatures (Fig. 2). Excluding the bias of alterations in neutrophil population size across conditions, gene set enrichment analysis on granulocyte samples now uncovered that differentially expressed genes between severe and mild COVID-19 patients are indeed characterized by an increase in granulocyte activation-associated factors (Additional file 2: Figure S5B). *CD177* is part of the granulocyte activation gene set and was indeed markedly increased in severe (day 1–10) compared to mild (day 1–10) COVID-19 samples (Fig. 3d). Also, the alarmin *S100A12* exhibited heightened expression in granulocytes from severe COVID-19 patients (Fig. 3d).

Next, we used the CoCena^2^ modules from the whole blood analysis (Fig. 2c) to identify modules that are actually driven by alterations in neutrophil activation instead of a mere increase in the neutrophil population. To investigate the expression patterns in a longitudinal fashion, mean expression over time and a confidence interval were calculated for each module in the mild and severe cases, respectively. Modules being mainly expressed in the severe groups G1 and G2 (darkgreen, darkgrey, lightgreen, maroon, and pink) showed a shift towards upregulation of genes in the severe group compared to the mild group, except for module darkgrey (Fig. 3e). The other modules, darkorange, gold, indianred, orchid, and steelblue, presented mostly the opposite trend, being expressed at higher levels in the mild compared to the severe COVID-19 cases (Additional file 2: Figure S5C).

Recently, heterogeneity of neutrophils with distinct subsets associated with disease severity and phase was revealed by single-cell RNA-seq analysis in blood of COVID-19 patients [34]. Enrichment of the three signatures that related to severe COVID-19 in our granulocyte samples demonstrated that the findings obtained in the single-cell study were also discernible in bulk data, and the results in accordance with the reported phenotypes: premature/immature, severe inflammatory, and severe suppressive subset marker genes were markedly enriched in granulocytes from severe COVID-19 patients in the present study (Additional file 2: Figure S5D). Further analysis of this observation on the gene level displayed the heightened expression of pre-/immature neutrophil-associated markers in severe COVID-19 patients’ granulocytes, such as FUT4 (CD15), metalloproteinase *MMP8*, alarmins (*S100A8/9*), NET formation-involved *PADI4*, or *NLRC4*, for which activating mutations have been reported to overtly trigger the inflammasome and thereby increase the risk to develop autoinflammatory syndrome [73, 74] (Fig. 3f). Marker genes attributed to the “mild mature activated” neutrophil subset [34], such as *ITGA4* or *SLC38A1*, were indeed elevated as well in the mild COVID-19 patients’ granulocytes of this study. In line with the single-cell study, signs of an interferon response were observed irrespective of disease severity (*IFIT1*, *IFIT3*), while only severe COVID-19 patients’ granulocytes featured expression of genes with suppressive functionality, such as *ARG1* or *CD274* (PD-L1) (Fig. 3f).

We next assessed the granulocyte samples based on the module signatures from the whole blood analysis. The granulocyte samples were filtered for the genes present in the COVID-19 co-expression network (Fig. 2c) and the group fold changes were calculated across all patients individually; sample groups G1–G5 were assigned according to their combinatorial module expression (Figs. 2c + 3g). For example, samples attributed to G1 showed high enrichment scores in modules lightgreen, darkgreen, and pink, whereas those assigned as G2 additionally expressed the maroon module. Samples with the indianred/darkorange combination were designated as G4. Re-analysis of *CD177*, *NLRC4*, *ARG1*, and *CD274* (PD-L1) as a function of the assigned sample groups (Fig. 3g) showed increased expression in G1 and G2 in relation to the other groups (Fig. 3h + Additional file 2: Figure S5E). Interestingly, the stratified patient groups in the whole blood data also depicted increased expression in G1 and G2 in comparison to the control-containing G6 (Fig. 3i + Additional file 2: Figure S5F).

Analysis of granulocyte samples from COVID-19 patients proved that, in addition to the relative increase in neutrophils in severe COVID-19 cases, there are indeed alterations in the transcriptional program of these cells themselves. We found enrichment of signatures typical of pre-/immature neutrophils and evidence of simultaneous inflammatory and suppressive features, arguing for a dysregulation in the peripheral granulocyte compartment. Importantly, transferring these findings back to the whole blood analysis showed that the granulocyte phenotypes were still observable within the whole blood transcriptomes.

### Integration with signatures from other diseases reveals COVID-19-specific characteristics

**Fig. 4:**
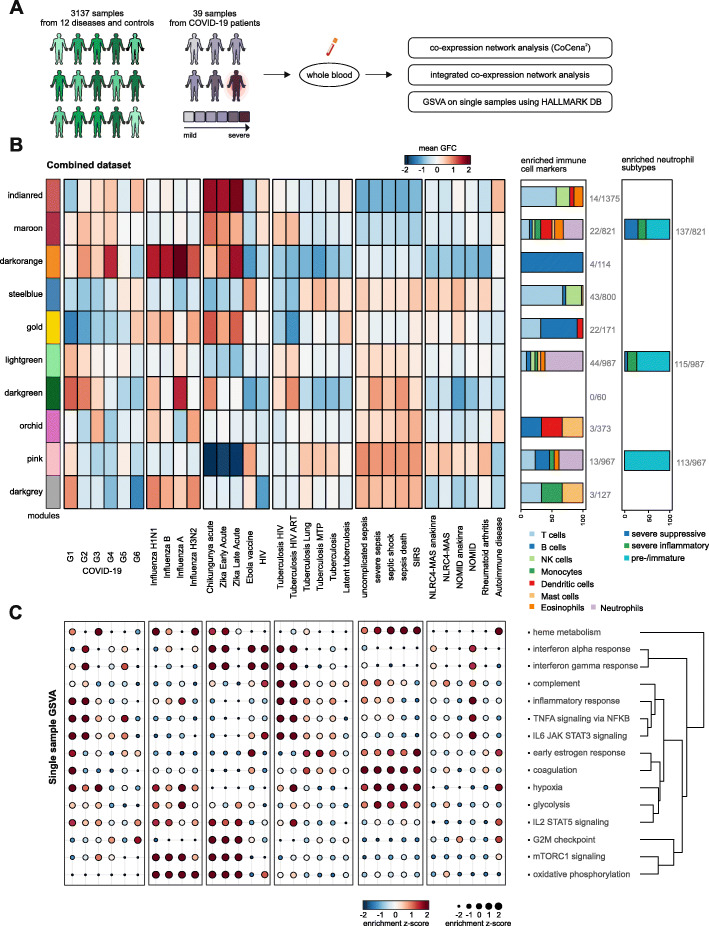
Integration with signatures from other diseases reveals COVID-19-specific characteristics. **a** Schema of analysis of the integrated dataset. The integrated dataset was analyzed with regard to expression patterns of the clusters previously identified in the whole blood COVID-19-specific co-expression network. **b** Heat map of mean group fold changes of CoCena^2^ module comparison between COVID-19 and other diseases. From left to right, the diseases are ordered by category (COVID-19, viral infections, bacterial infections, and others). On the right side of the heat map, the first box plot shows the enriched immune cell markers in each module. The second box plot shows the enrichment of genes upregulated in specific neutrophil subtypes based on cross-referencing with single-cell data [34]. Both box plots show enriched cell types in percent of total hits; absolute hits with respect to cluster size are stated aside. **c** Gene set variation analysis was conducted for every single patient based on Hallmark gene sets as shown in Fig. 2d. The result was standardized to retrieve the *z*-scores; a disease mean was calculated and displayed as a dot plot with size and color correlating to the *z*-score. The labels on the *x*-axis are the same as in **b**

Putting COVID-19 into context of other known diseases, we compiled whole blood transcriptomes from 12 further diseases, including several viral and bacterial infections as well as immune-related disorders into one large dataset encompassing a total of 3176 samples including the 39 COVID-19 samples from this study (Fig. 4a, Additional file 2: Figure S6A, Additional file 6: Table S5). All in all, the dataset contains four other viral infection studies (chikungunya [30], HIV [27], influenza [75], and Zika [76], *n* = 695), seven bacterial infection studies (tuberculosis [24–27, 77], bacterial sepsis and systemic inflammatory response syndrome (SIRS, *n* = 1578) [28]), six inflammatory/autoimmune studies (systemic lupus erythematosus [78], Crohn’s disease, rheumatoid arthritis [79], Ebola vaccination [29], neonatal-onset multisystem inflammatory disease (NOMID), and macrophage activation syndrome (NLRC4-MAS) [74], *n* = 326), and control samples from nine different studies (*n* = 538). To investigate how the COVID-19-specific co-expression modules can be linked to other diseases, the combined dataset was filtered for the genes present in the COVID-19 co-expression network (Fig. 2c) and the group fold changes were calculated across all samples (Fig. 4b). Additionally, cell type-specific signatures [50] and single cell-derived neutrophil subset signatures [34] (Additional file 7: Table S6) were intersected with all CoCena^2^ modules. This analysis revealed that the lightgreen module shows a high (61%) neutrophil enrichment followed by module pink (38%) and maroon (32%), which is in line with a high functional enrichment for neutrophil activation in lightgreen (Fig. 2e, Additional file 4: Table S3). Genes within module lightgreen were most prominently upregulated in the severe COVID-19 group (G1) as well as in sepsis, in patients with influenza A and with tuberculosis and HIV infection, but less so in individually occurring HIV and tuberculosis (Fig. 4b). Enrichment of the neutrophil subset signatures revealed increased expression of genes found in pre-/immature neutrophils and those of inflammatory neutrophils associated with severe COVID-19. Many genes within module lightgreen are known to be related to induction of neutrophil extracellular traps (NET) (e.g., *PKC* [80], *PADI4* [81], *LTB4* [82]). Moreover, a link between excessive NET formation and tissue damage has been reported in sepsis [83]. Module darkgrey shares a similar expression pattern across the disease spectrum with lightgreen though being upregulated in infection with any of the four included influenza strains and contains genes involved in platelet activation. The NET-platelet-thrombin axis has been reported to be involved in the promotion of intravascular coagulation in sepsis [84]. The pink module shows the second highest neutrophil enrichment, which is dominated by the enrichment of pre-/immature neutrophil subtype signatures. It is strongly increased in sepsis, tuberculosis, and after Ebola vaccination as well as in autoinflammatory diseases such as rheumatoid arthritis, NLRC4-MAS, and NOMID, and shows slight overlap with the severe COVID-19 patients in group G1. It contains many epigenetic modifiers, such as *HDAC5*, *SETD1B*, or *KMT2D*, as well as *KLF2*, shown to regulate NF-κB-mediated immune functions, such as inflammation, erythropoiesis, and lung development [85]. Maroon is the third module with predicted neutrophil enrichment, which features genes from the “severe suppressive” subset alongside the “severe inflammatory” and pre-/immature subset signatures. It is associated with COVID-19 groups G2–4 and shares this characteristic with blood transcriptomes from the response to infection with chikungunya and Zika virus or from HIV patients suffering from tuberculosis.

A combination of single sample gene set variation analysis (ssGSVA), a non-parametric, unsupervised approach to estimate variation of gene set enrichment within each single sample, and Hallmark enrichment for each disease or inflammatory condition in the compiled dataset accentuated the findings on COVID-19 blood transcriptomes in context of the other diseases (Fig. 4c). “Interferon alpha and gamma responses” were enriched in acute viral infections with chikungunya and Zika virus as well as in HIV with or without concomitant tuberculosis or after Ebola vaccination, and this enrichment was shared with COVID-19 G2. “Inflammatory response,” “IL6 and TNFA signaling” is an attribute of both G1 and G2, to a lesser degree of G5, also tuberculosis/HIV, and to some extent of sepsis and influenza A. More prominently enriched in sepsis were “complement,” “coagulation,” “heme metabolism,” and “glycolysis” — shared by COVID-19 G1+G3, whereas “oxidative phosphorylation” and “mTORC1 signaling” were seen for all four influenza strains, chikungunya, and Zika virus infections — shared to some extent with COVID-19 G3+G4.

Although we observed overlaps of gene modules enriched in COVID-19 with several other infectious and immune-related diseases, each of our molecularly defined COVID-19 patient groups was characterized by a specific combination of these modules, clearly indicating the unique biology of this SARS-CoV-2 infection-mediated immune response, which needs to be considered when developing patient-stratified therapy regimens.

### COVID-19 patient subgroup-specific signatures can be used to predict potential drug repurposing

Despite the immunologically driven nature of COVID-19, most drugs that are currently investigated in clinical trials to combat or ameliorate COVID-19 are targeting the virus and its direct interaction partners (Additional file 2: Figure S7A+B, Additional file 8: Table S7). Compounds as well as the number of clinical trials performed with anti-inflammatory, immunosuppressive, and immunomodulatory properties are immensely outnumbered by other approaches. Examining the listed target genes of currently investigated drugs in our stratified patient groups, we found 162 included in our co-expression network analysis, most of which being differentially expressed in the severe patient group G1 in comparison to G6 (Figs. 2c + 5a). In addition, many of the regulated genes in our patient signatures are clearly not affected by the drugs that are currently investigated against COVID-19. The immunopathologies seen in COVID-19 patients, especially past their second week of symptoms, demand a host-directed, immune system-focused therapy.

**Fig. 5:**
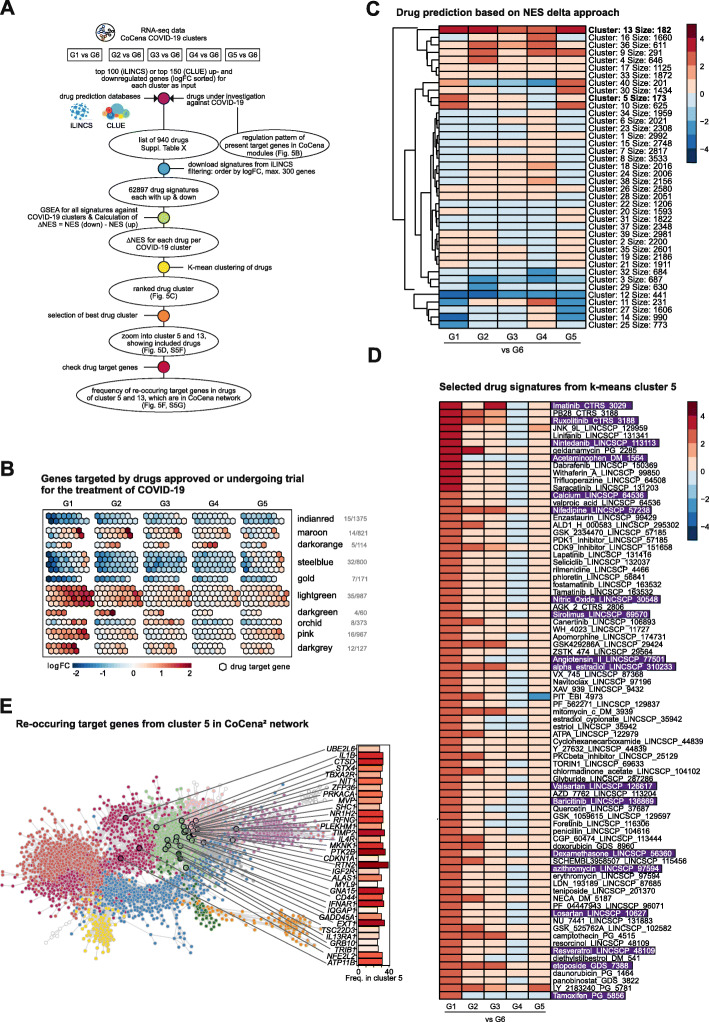
Patient subgroup-specific signatures can be used to predict potential drug targets. **a** Schematic workflow of the drug prediction analysis. Drug signatures were collected using the platforms iLINCS and CLUE. Signatures were selected by highest counteracting ΔNES score and combined with signatures of drugs under investigation from the literature. **b** Visualization of genes targeted by drugs approved or undergoing trial for the treatment of COVID-19 patients included in the whole blood co-expression network. Numbers of such genes from each module are designated on the right of the panel. Genes are represented as hexagons and colored by the expression fold change between COVID-19 patient severity group (G1–G5) and the control group (G6) (upregulated: red, downregulated: blue, not regulated: grey). **c** Drug predictions based on ΔNES score of drug signatures in regard to diseased patient group-specific gene expression patterns (G1–5 vs G6). Signatures were clustered by *k*-means clustering. A high ΔNES score accounts for drug signatures which counteract the gene expression of the patient group they are compared to. Drug signatures with a negative ΔNES score induce a gene expression pattern similar to the input. The number of signatures within a cluster determines its size. **d** Display of selected drug signatures from *k*-means cluster 5 from **c** showing the highest ΔNES score in the most severe COVID-19 patient group G1 and the least effect in patient group G4. **e** Visualization of recurring target genes in the G1 vs G6 comparison of cluster 5 signatures and their frequency mapped onto the CoCena^2^ network

To identify potentially beneficial drugs, we designed an in silico signature-based drug repurposing approach (Fig. 5b). To generate input signatures of interest, we characterized our stratified sample groups by identifying differentially expressed genes between groups G1–G5 and the control group G6 (Additional file 2: Figure S7C). Most transcriptional differences were observed for G1 (up: 4032, down: 4729) and G2 (up: 2336, down: 2767), whereas group G3 (up: 1193, down: 1921), G5 (up: 1089, down: 1216), and especially G4 (up: 727, down: 547) were less different to G6. Only a minor fraction of 137 DEGs was shared by all 5 comparisons. The most overlap of DEGs was observed between G1 and G2, the two groups comprising mostly severe COVID-19 patients. Nevertheless, G2 was still characterized by a large number of specific DEGs (Additional file 2: Figure S7C). GOEA of the upregulated DEGs of each comparison revealed enrichment of genes in the context of “neutrophil activation” and “coagulation” in all comparisons (Additional file 2: Figure S7D). Humoral and B cell-mediated immunity terms on the other hand were enriched the strongest in G4-specific upregulated DEGs (Additional file 2: Figure S7D). Differential expression analysis for the stratified sample groups once more emphasized that neutrophils play a central role in the host’s immune response against SARS-CoV-2 infection. Neutrophils, as the most abundant circulating leukocytes, have become a therapeutic target of interest in multiple disease settings in recent years [86]. Two interesting target genes discussed in this context and already addressed in clinical trials are CXCR2 and C5AR1. Consistent with the increased NLR in G1 and G2, we observed significant upregulation of *CXCR2* and *C5AR1* in both groups (Additional file 2: Figure S7E).

Using patient cluster-specific DEGs as input (Additional file 2: Figure S7C, Additional file 9: Table S8), we searched for compounds that evoke a reverse signature in human cells via the NIH Library of Integrated Network-Based Cellular Signatures (iLINCS) [87] and the Broad Institute’s Repurposing Hub [88]. The best counteracting signatures for each comparison were combined with signatures for all currently investigated drugs and downloaded for further analysis, resulting in about 63,000 signatures from 940 compounds/drugs. We performed gene set enrichment analysis for all signatures against our COVID-19 patient comparisons and calculated the difference of the up- and downregulated normalized enrichment score (ΔNES). A positive ΔNES indicates drug signatures that reverse our COVID-19 signatures, whereas drugs with a negative ΔNES induce signatures similar to the ones observed in COVID-19. Signatures were then grouped by *k*-means clustering revealing groups of drug signatures that reverse specific patient subgroup signatures (e.g., cluster 5) or those that have the highest impact on all patient subgroups (e.g., cluster 13, Fig. 5c). Among the top signatures in cluster 13 are methylprednisolone (ΔNES_G1_ = 7.13), immunoglobulins (ΔNES_G1_ = 6.62), methotrexate (ΔNES_G1_ = 4.21), and pevonedistat (ΔNES_G1_ = 4.81) which are all under investigation (clinicaltrials.gov), thereby proving that our in silico signature-based drug repurposing approach can indeed predict drugs that have already been deemed potentially beneficial in this disease (Additional file 2: Figure S7F). Extracting the leading edge of the most frequently targeted genes by the drugs included in cluster 13 revealed alarmins, such as *S100A8* or *S100A6*, and *SERPINB1*, critical for neutrophil survival by protecting the cell from proteases released into the cytoplasm during stress [89–91]. Visualizing these genes in the co-expression network deducted from the blood transcriptomes of our COVID-19 patient cohort identified most of them as part of cluster lightgreen and maroon (Additional file 2: Figure S7G). Sample group G1-specific drug signature cluster 5 also encompasses a considerable number of drugs currently being tested in clinical trials to fight COVID-19 (Fig. 5d + Additional file 2: Figure S7A, Additional file 10: Table S9). Interestingly, a lot of drug signatures in cluster 5 were related to female hormones, such as alpha-estradiol (ΔNES_G1_ = 2.83), estradiol-cypionate (ΔNES_G1_ = 2.78), estriol (ΔNES_G1_ = 2.78), or chlormadinone acetate used in birth control pills (ΔNES_G1_ = 2.74), but also for example dexamethasone (ΔNES_G1_ = 2.65) that was recently reported to reduce mortality in severe COVID-19 cases requiring intubation, while showing no benefit for patients with milder disease courses [92]. The most frequently targeted genes within the signatures of cluster 5 included protein tyrosine kinase 2 beta (*PTK2B*), playing an important role for integrin-mediated neutrophil degranulation [93, 94]; lysosomal protease cathepsin D (*CTSD*) expressed in neutrophils and monocytes; and the inflammatory mediator interleukin-1β (*IL1B*) (Fig. 5e). The majority of these target genes cluster in the G1-specific lightgreen and pink, as well as in the maroon CoCena^2^ modules. Drugs predicted to be effective for each module are presented as a resource as supplementary information for further inspection (Additional file 10: Table S9).

We used stratified blood transcriptomes from COVID-19 patients in an in silico signature-based approach to identify potential drugs for therapeutic repurposing. Many of our identified hits are indeed already being tested in clinical trials. Further, it became evident that, apart from common therapeutic avenues to address the immune dysregulation in COVID-19 patients, there are patient groups that may benefit from treatments targeting more precisely their immune phenotype and this phenotyping could be used for enrichment of patient groups in clinical trials.

## Discussion

The global spread of SARS-CoV-2 resulting in hundreds of thousands of COVID-19 cases urgently demands a more thorough molecular understanding of the pathophysiology of the disease [15, 20, 95, 96]. While vaccines are still under development [97–102], therapeutic management of the COVID-19 patients is key to mitigate the clinical burden as well as to prevent deaths. It has become clear that there is great variety in the occurrence of disease manifestation, and dysregulation of local and systemic immune responses have been implicated in disease heterogeneity [12–14, 22, 37, 38, 42, 95, 103, 104]. Here, by applying classical bioinformatics approaches and data-driven co-expression network analysis (CoCena^2^) on blood transcriptomes of COVID-19 patients, we provide evidence for the existence of distinct molecular phenotypes that are not solely explained by current clinical and immunological parameters. Particularly in severe COVID-19, we detected dramatic transcriptional changes in the blood compartment with loss of T cell activation and concurrent gain of a rather unique combination of neutrophil activation signals, which was not simply due to changes in cell numbers since isolated neutrophils showed the same transcriptional changes. CoCena^2^ allowed us to group functionally related genes into 10 major transcriptional modules with distinct expression patterns across five, on this basis newly defined COVID-19 patient groups, of which two (G1, G2) were related to severe disease courses. While pronounced neutrophil-related alterations were observed in both subgroups of severe COVID-19 patients (G1, G2), genes associated with coagulation and platelet function were mainly elevated in patients with the most highly elevated number of neutrophils as measured by flow cytometry, an information that was also deduced by linear support vector regression from transcriptome data. Assessment of non-coding RNA species from whole blood transcriptomes also allowed the identification for additional regulatory circuits. For example, we identify *CYTOR*, a lncRNA associated with granulocyte survival [59] strongly upregulated in COVID-19 patient group G1, which was accompanied by strong induction of CYTOR interactors such as *VIM* and *PIK3CB* [69]. These findings strongly support the notion that whole blood transcriptomics might not only be suitable for better understanding the systemic immune response in COVID-19 patients, but can also be used to predict novel therapeutic targets involving distinct pathophysiological mechanisms observed in COVID-19. In a “reverse transcriptome approach,” we used the specific changes observed in the COVID-19-related transcriptional modules as the bait and searched for inverse correlation in thousands of drug-based transcriptome signatures to predict potential drug candidates. Most interestingly, we identified drug candidates that might be beneficial for all COVID-19 patients, but also candidates that might only be suitable for a subgroup of patients. Lastly, by comparing the transcriptional modules identified in whole blood of COVID-19 patients, we identified unique differences to other viral and bacterial infections, for which similar data were available, suggesting that blood transcriptomes might also be used diagnostically or for outcome prediction in larger clinical cohorts, treatment, or vaccine trials in the near future.

Classical bioinformatic assessment of blood transcriptome data comparing defined groups, in this study represented by control individuals and samples derived from either mild or severe COVID-19 patients, already revealed important biology of the systemic immune response. For example, the most significantly elevated transcript was *CD177*, a cell surface molecule on neutrophils, which was enhanced in both mild and severe cases (Fig. 1, Additional file 2: Figure S1), was recently identified by proteomics in bronchoalveolar lavage of COVID-19 patients [105], and has also been introduced as a hallmark for Kawasaki syndrome [106], a syndrome that has been observed in several studies being increased in children and adolescents during the SARS-CoV-2 pandemic [107–112]. In acute Kawasaki syndrome, elevated expression of *CD177* was associated with resistance to treatment with intravenous immunoglobulin (IVIG), a therapy in COVID-19 patients that is currently investigated in clinical trials around the world (18 trials, clinicaltrials.gov). Integrating the assessment of CD177 into these trials might help to stratify patients and better predict individual therapy outcome.

Hierarchical clustering of the most variable genes in the dataset already hinted towards further heterogeneity among patients beyond the current clinical differentiation into mild and severe patients (Fig. 1). Indeed, co-expression network analysis in a data-driven fashion allowed us to define five patient subgroups (G1–5) defined by 10 distinct transcriptional modules, which was corroborated in a second independent cohort (Fig. 2 + Additional file 2: Figure S4). Gene transcription observed in severe COVID-19 patients in G1 clearly differed from severe G2 COVID-19 patients particularly in modules darkgrey, pink, orchid, and maroon (Fig. 2c). For example, biological mechanisms related to the darkgrey module included blood coagulation, platelet activation, aggregation, and degranulation, as well as cell-cell adhesion and integrin-mediated signaling. These are all mechanisms that are integral to several of the complications observed in a subset of severe COVID-19 patients including increased disseminated intravascular coagulation [113–115], venous thromboembolism [113, 116], stroke [117, 118], or acute cor pulmonale [119]; neutrophil extracellular traps have been reported to contribute to immunothrombosis seen in pulmonary autopsies [120, 121]. All in all, these findings support the need for advanced molecular subtyping of COVID-19 patients, as proposed here based on blood transcriptomes. This is only one prominent example of the rich information within the new structure of molecular COVID-19 phenotypes that we provide here. For further inspection of the data, we refer the reader to the online tool that allows to extract module and group specific gene expression information (https://www.fastgenomics.org/).

In addition to many other infectious and non-infectious diseases [24–32], whole blood transcriptomics revealed important insights into the patient structure in COVID-19 and comparative analysis provides first evidence for the unique changes elicited by this disease within the host in comparison to other infections (Fig. 4). While cases in G2–4 shared changes with other viral infections such as influenza, chikungunya, or Zika, mainly including interferon signature genes (*IFI16*, *IFI35*, *IFIT1*, maroon module), partial overlap to bacterial sepsis was observed for G1–G3, albeit the major sepsis module (pink) was not prominently enriched in COVID-19 patients indicating that there are distinct differences in pathology of these two diseases. Although we could establish an integrative model using historical and publicly available blood transcriptome data, we also realized that limited standardization of the experimental procedures (sample processing, library production, sequencing) between different whole blood transcriptomics studies led to the exclusion of several additional important studies. In this context, it will be of great interest whether blood transcriptomics, as it was shown for tuberculosis [24, 25], can be utilized in large enough cohorts and clinical trials for disease risk or outcome prediction in COVID-19. We propose to collect whole blood transcriptomics data in a central registry for direct inspection by the research community and provide a prototype model for such a registry on FASTGenomics. Transcriptome data have been successfully used to predict a role for specific gene networks in the drug response to certain cancer types [122–126]. Considering the strong influence of the systemic immune response on severity and outcome of COVID-19, we wanted to establish whether the global assessment of molecular subgroups of COVID-19 patients could be utilized to predict novel drug targets for this disease addressing the dysregulated peripheral immune response of the host (Fig. 5). Using two major databases providing transcriptome signatures to many known drugs, CLUE [126] and iLINCS [125], we designed an in silico signature-based drug repurposing approach, allowing us to identify candidate drugs [127] that might reverse immune pathophysiology as observed in blood transcriptomes. Some of the candidate drugs identified are currently already in clinical trials, for example imatinib (NCT04394416, NCT04357613, NCT04346147, NCT04422678), ruxolitinib (20 trials listed), or nintedanib (NCT04338802, NCT04541680), for which prediction was particularly high in G1 patients. These trials might benefit from assessing molecular phenotypes of immune cells thereby determining whether patients with G1 type transcriptomes benefit most from such treatment. First study reports have recently declared strong benefit for dexamethasone treatment in severe COVID-19 cases requiring intubation, while no effect on mortality was seen for those patients who did not require respiratory support [23, 92]. Of note, drugs predicted to potentially reverse the transcriptome signatures of the severely affected G1 group may have adverse effects in milder COVID-19 cases from G4 as observed in the contrasting regulation patterns in many of the clusters (Fig. 5c). Interestingly and in line with the reports on sexual dimorphism in COVID-19 severity and mortality [128], G1 included only male patients and many of the drugs predicted to reverse the G1-specific signatures were related to female hormones (Fig. 5d). However, we also predicted drugs for all COVID-19 patients already in clinical trials such as immunoglobulins (> 150 trials, clinicaltrials.gov), or methylprednisolone (19 trials), findings further supporting the value of our prediction approach. Despite these promising results, strongly suggesting that reverse transcriptomics is not only of value in cancer [122–124] but might also be used to identify drugs targeting the immune pathophysiology in COVID-19, we would also like to point out current limitations of our findings that need to be addressed in future studies. Predictions, as well as also the molecular phenotypes for patient stratification, will further benefit from and focused by validation studies in independent COVID-19 patient cohorts, which is to be fostered by a central database for COVID-19 patients’ blood transcriptome data. These additional studies will also be able to further address disease severity in combination with different patient demographics and additional clinical parameters. Nevertheless, we used samples from different countries, illustrating the generalizability. Furthermore, the molecularly derived and prioritized drug candidates presented here might be tested in very recently introduced pre-clinical models [129] prior to starting clinical trials. Irrespective of the current shortcomings, we favor such drug candidate identification, since it is based on interrogation of molecular data directly derived from patients’ immune cells involved in the ongoing processes in the disease and therefore may increase the likelihood of a beneficial effect in patients.

## Conclusions

Collectively, we provide first evidence for whole blood transcriptomics to potentially become a valuable tool for distinguishing the peripheral immune response seen in COVID-19 from that in other infections in cases for which pathogen detection might be difficult, for monitoring and potentially predicting outcome of the disease, to further dissect molecular phenotypes of COVID-19, particularly of the host’s immune system, also along the disease course over time, and to support drug target prediction for subgroups of patients. Clearly, in contrast to more sophisticated higher resolution methods, whole blood transcriptomes can be easily obtained in large clinical cohort studies and large clinical treatment trials yet providing an enormous information content about the molecular reactions of the host’s immune system. We therefore propose a blood transcriptome registry following the model we introduce here on the FASTGenomics platform that would allow the scientific community to utilize the information for new clinical studies and to address further large-scale studies into pathophysiological mechanisms of the disease and enhance the chances of trials to demonstrate a clinical benefit in patients.

## Supplementary Information

**Additional file 1: Table S1.** Cohort statistics.

**Additional file 2: Supplementary Figures S1-S8**.

**Additional file 3: Table S2.** Differential genes.

**Additional file 4: Table S3.** CoCena modules.

**Additional file 5: Table S4.** Granulocytes anno, differential genes and neutrophil subtypes.

**Additional file 6: Table S5.** Overview of datasets.

**Additional file 7: Table S6.** Immune cell type signatures.

**Additional file 8: Table S7.** Drugs overview.

**Additional file 9: Table S8.** Differential genes of groups G1-G6.

**Additional file 10: Table S9.** Predicted drugs.

## Data Availability

The data that support the findings of this study, including transcriptome data from 95 patients (123 samples) at multiple time points who granted informed consent to share such data, are made available at the European Genome-Phenome Archive (EGA) under accession number EGAS00001004503, which is hosted by the EBI and the CRG. The Rhineland Study’s dataset is not publicly available because of data protection regulations. Access to data can be provided to scientists in accordance with the Rhineland Study’s Data Use and Access Policy. Requests for further information or to access the Rhineland Study’s dataset should be directed to RS-DUAC@dzne.de. All scripts and all processed data are available under https://github.com/schultzelab/COVID-19-blood-bulk-RNA-Seq [130]. In addition to data deposition on EGA and Github, we provide an interactive platform for data inspection and analysis via FASTGenomics (fastgenomics.org). The FASTGenomics platform also provides normalized count tables of the datasets generated in this study. CoCena^2^ is also available under https://github.com/Ulas-lab/CoCena2 [131]. The publicly available datasets analyzed during the current study are available from the *EGA repository* https://ega-archive.org/studies/EGAS00001004503 [132] *GEO repository* GSE111368 (https://www.ncbi.nlm.nih.gov/geo/query/acc.cgi?acc=GSE111368) [75] GSE101705 (https://www.ncbi.nlm.nih.gov/geo/query/acc.cgi?acc=GSE101705) [26] GSE107104 (https://www.ncbi.nlm.nih.gov/geo/query/acc.cgi?acc=GSE107104) [27] GSE112087 (https://www.ncbi.nlm.nih.gov/geo/query/acc.cgi?acc=GSE112087) [78] GSE127792 (https://www.ncbi.nlm.nih.gov/geo/query/acc.cgi?acc=GSE127792) [133] GSE128078 (https://www.ncbi.nlm.nih.gov/geo/query/acc.cgi?acc=GSE128078) [134] GSE129882 (https://www.ncbi.nlm.nih.gov/geo/query/acc.cgi?acc=GSE129882) [76] GSE133378 (https://www.ncbi.nlm.nih.gov/geo/query/acc.cgi?acc=GSE133378) [32] GSE143507 (https://www.ncbi.nlm.nih.gov/geo/query/acc.cgi?acc=GSE143507) GSE57253 (https://www.ncbi.nlm.nih.gov/geo/query/acc.cgi?acc=GSE57253) [74] GSE63042 (https://www.ncbi.nlm.nih.gov/geo/query/acc.cgi?acc=GSE63042) [28] GSE66573 (https://www.ncbi.nlm.nih.gov/geo/query/acc.cgi?acc=GSE66573) [135] GSE79362 (https://www.ncbi.nlm.nih.gov/geo/query/acc.cgi?acc=GSE79362) [24] GSE84076 (https://www.ncbi.nlm.nih.gov/geo/query/acc.cgi?acc=GSE84076) [77] GSE89403 (https://www.ncbi.nlm.nih.gov/geo/query/acc.cgi?acc=GSE89403) [25] GSE90081 (https://www.ncbi.nlm.nih.gov/geo/query/acc.cgi?acc=GSE90081) [79] GSE97590 (https://www.ncbi.nlm.nih.gov/geo/query/acc.cgi?acc=GSE97590) [29] GSE99992 (https://www.ncbi.nlm.nih.gov/geo/query/acc.cgi?acc=GSE99992) [30] *BioProject repository* PRJNA588242 (https://www.ncbi.nlm.nih.gov/bioproject/PRJNA588242) [136].
