# Supplementary material for: Disease severity-specific neutrophil signatures in blood transcriptomes stratify COVID-19 patients
Source: Genome Med. 2021 Jan 13;13:7. doi: 10.1186/s13073-020-00823-5 (PMC7805430; doi:10.1186/s13073-020-00823-5)
Supplement: Supplementary file 2 — Additional file 2: Supplementary Figures S1-S8. [file 13073_2020_823_MOESM2_ESM.pdf]

Fig. S1

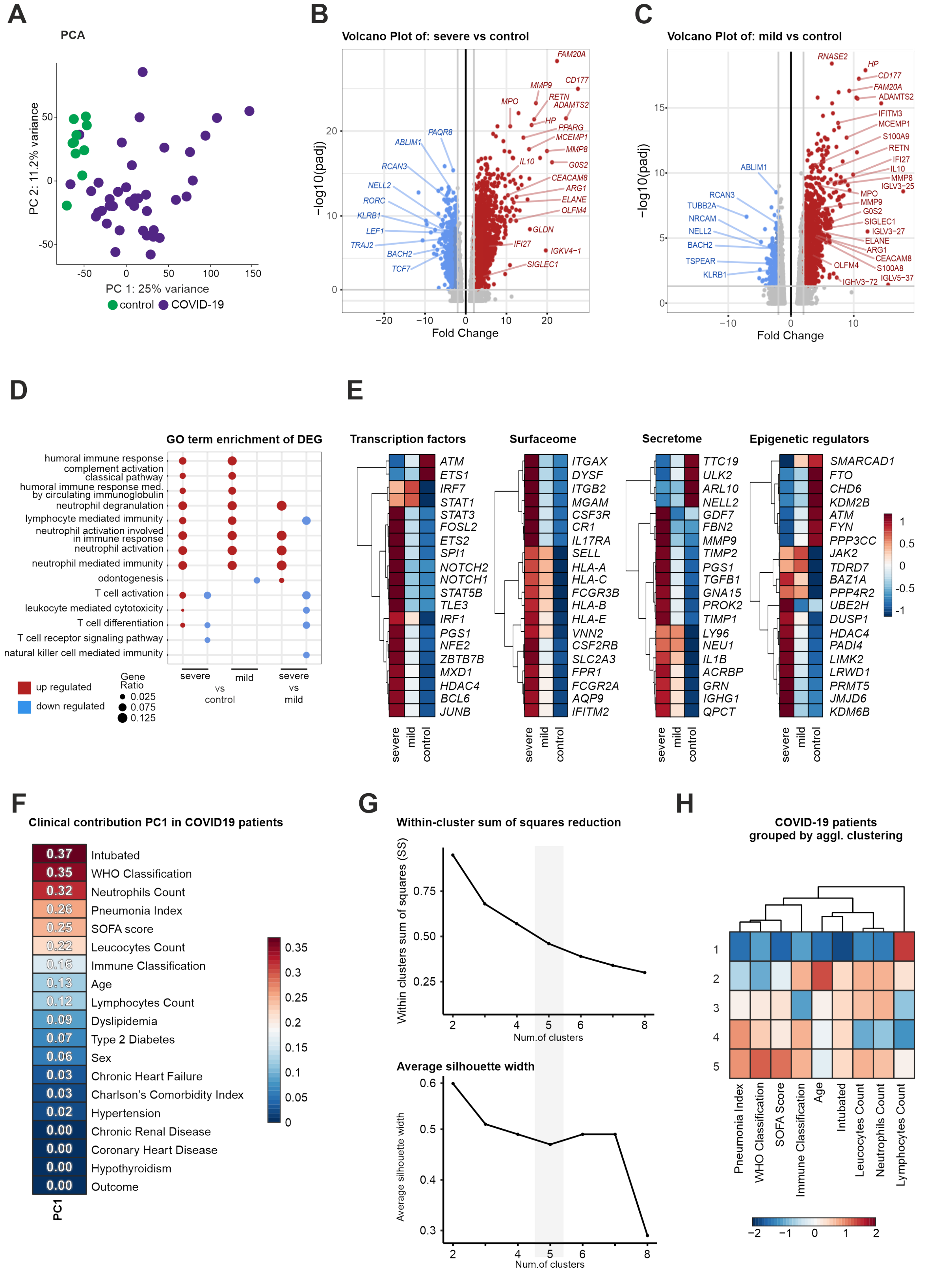

A
PCA
PC 2: 11.2% variance
PC 1: 25% variance
control
COVID-19
B
Volcano Plot of: severe vs control
-log10(padj)
Fold Change
FAM20A
CD177
MMP9
RETN
ADAMTS2
MPO
HP
PPARG
MCEMP1
MMP8
IL10
G0S2
CEACAM8
ARG1
ELANE
OLFM4
GLDN
IFI27
IGKV4-1
SIGLEC1
PAQR8
ABLIM1
RCAN3
NELL2
RORC
KLRB1
LEF1
TRAJ2
BACH2
TCF7
C
Volcano Plot of: mild vs control
-log10(padj)
Fold Change
RNASE2
HP
CD177
FAM20A
ADAMTS2
IFITM3
MCEMP1
S100A9
RETN
IFI27
IL10
MMP8
IGLV3-25
MPO
MMP9
G0S2
SIGLEC1
IGLV3-27
ELANE
ARG1
CEACAM8
S100A8
IGLV5-37
IGHV3-72
OLFM4
ABLIM1
RCAN3
TUBB2A
NRCAM
NELL2
BACH2
TSPEAR
KLRB1
D
GO term enrichment of DEG
humoral immune response
complement activation classical pathway
humoral immune response med. by circulating immunoglobulin
neutrophil degranulation
lymphocyte mediated immunity
neutrophil activation involved in immune response
neutrophil activation
neutrophil mediated immunity
odontogenesis
T cell activation
leukocyte mediated cytotoxicity
T cell differentiation
T cell receptor signaling pathway
natural killer cell mediated immunity
up regulated
down regulated
Gene Ratio
0.025
0.075
0.125
severe
mild
vs
control
severe
vs
mild
E
Transcription factors
ATM
ETS1
IRF7
STAT1
STAT3
FOSL2
ETS2
SPI1
NOTCH2
NOTCH1
STAT5B
TLE3
IRF1
PGS1
NFE2
ZBTB7B
MXD1
HDAC4
BCL6
JUNB
Surfaceome
ITGAX
DYSF
ITGB2
MGAM
CSF3R
CR1
IL17RA
SELL
HLA-A
HLA-C
FCGR3B
HLA-B
HLA-E
VNN2
CSF2RB
SLC2A3
FPR1
FCGR2A
AQP9
IFITM2
Secretome
TTC19
ULK2
ARL10
NELL2
GDF7
FBN2
MMP9
TIMP2
PGS1
TGFB1
GNA15
PROK2
TIMP1
LY96
NEU1
IL1B
ACRBP
GRN
IGHG1
QPCT
Epigenetic regulators
SMARCAD1
FTO
CHD6
KDM2B
ATM
FYN
PPP3CC
JAK2
TDRD7
BAZ1A
PPP4R2
UBE2H
DUSP1
HDAC4
PADI4
LIMK2
LRWD1
PRMT5
JMJD6
KDM6B
severe
mild
control
F
Clinical contribution PC1 in COVID19 patients
0.37 Intubated
0.35 WHO Classification
0.32 Neutrophils Count
0.26 Pneumonia Index
0.25 SOFA score
0.22 Leucocytes Count
0.16 Immune Classification
0.13 Age
0.12 Lymphocytes Count
0.09 Dyslipidemia
0.07 Type 2 Diabetes
0.06 Sex
0.03 Chronic Heart Failure
0.03 Charlson's Comorbidity Index
0.02 Hypertension
0.00 Chronic Renal Disease
0.00 Coronary Heart Disease
0.00 Hypothyroidism
0.00 Outcome
PC1
G
Within-cluster sum of squares reduction
Within clusters sum of squares (SS)
Num.of clusters
Average silhouette width
Average silhouette width
Num.of clusters
H
COVID-19 patients grouped by aggl. clustering
Pneumonia Index
WHO Classification
SOFA Score
Immune Classification
Age
Intubated
Leucocytes Count
Neutrophils Count
Lymphocytes Count

Figure S1 – related to Figure 1

(A) PCA plot depicting relationship of all samples based on dynamic gene expression of all genes comparing COVID-19 and control samples.

(B-C) Volcano plots depicting fold changes and FDR-adjusted p-values comparing severe **(A)** or mild **(B)** COVID-19 patients vs. controls. Differentially expressed up- (red) and downregulated genes (blue) are shown and selected genes are highlighted.

(D) Plot of top 10 most enriched GO terms for significantly up- and downregulated genes. Ratios of significantly regulated genes within enriched GO terms (GeneRatio) are shown for the comparisons between mild or severe COVID-19 patients and controls as well as between 'mild' and 'severe' COVID-19 patients.

(E) Heat map of group mean gene expression values from the top 20 most variant transcription factors, epigenetic regulators, surface and secreted proteins.

(F) Heat map of the linear model adjusted r-square that includes each clinical parameter with PC1. Clinical parameters with r-adjusted square ≥0.1 were used for agglomerative clustering of COVID-19 patients.

(G) Plots for agglomerative clustering statistics: within cluster sum of squares and high average silhouette width scores.

(H) Heat map presenting summary statistics of the clinical parameters used for the clustering across clinical agglomerative clusters 1-5.

Fig. S2

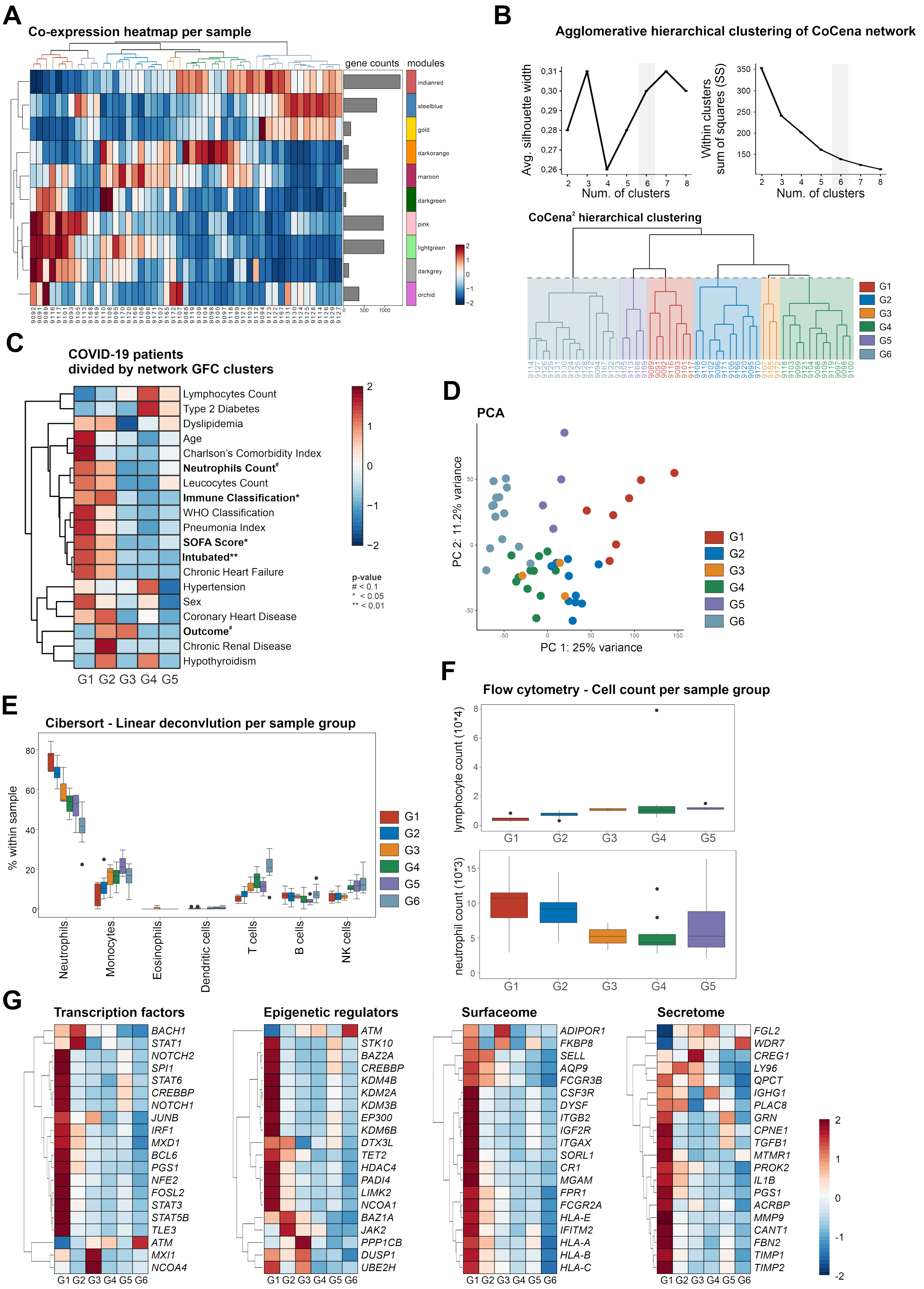
A
Co-expression heatmap per sample
gene counts
modules
indianred
steelblue
gold
darkorange
maroon
darkgreen
pink
lightgreen
darkgrey
orchid
B
Agglomerative hierarchical clustering of CoCena network
Avg. silhouette width
Num. of clusters
Within clusters sum of squares (SS)
Num. of clusters
CoCena² hierarchical clustering
G1
G2
G3
G4
G5
G6
C
COVID-19 patients divided by network GFC clusters
Lymphocytes Count
Type 2 Diabetes
Dyslipidemia
Age
Charlson's Comorbidity Index
Neutrophils Count#
Leucocytes Count
Immune Classification*
WHO Classification
Pneumonia Index
SOFA Score*
Intubated**
Chronic Heart Failure
Hypertension
Sex
Coronary Heart Disease
Outcome#
Chronic Renal Disease
Hypothyroidism
G1 G2 G3 G4 G5
p-value
< 0.1
* < 0.05
** < 0.01
D
PCA
PC 2: 11.2% variance
PC 1: 25% variance
E
Cibersort - Linear deconvlution per sample group
% within sample
Neutrophils
Monocytes
Eosinophils
Dendritic cells
T cells
B cells
NK cells
F
Flow cytometry - Cell count per sample group
lymphocyte count (10^4)
neutrophil count (10^3)
G
Transcription factors
BACH1
STAT1
NOTCH2
SPI1
STAT6
CREBBP
NOTCH1
JUNB
IRF1
MXD1
BCL6
PGS1
NFE2
FOSL2
STAT3
STAT5B
TLE3
ATM
MXI1
NCOA4
Epigenetic regulators
ATM
STK10
BAZ2A
CREBBP
KDM4B
KDM2A
KDM3B
EP300
KDM6B
DTX3L
TET2
HDAC4
PADI4
LIMK2
NCOA1
BAZ1A
JAK2
PPP1CB
DUSP1
UBE2H
Surfaceome
ADIPOR1
FKBP8
SELL
AQP9
FCGR3B
CSF3R
DYSF
ITGB2
IGF2R
ITGAX
SORL1
CR1
MGAM
FPR1
FCGR2A
HLA-E
IFITM2
HLA-A
HLA-B
HLA-C
Secretome
FGL2
WDR7
CREG1
LY96
QPCT
IGHG1
PLAC8
GRN
CPNE1
TGFB1
MTMR1
PROK2
IL1B
PGS1
ACRBP
MMP9
CANT1
FBN2
TIMP1
TIMP2
G1 G2 G3 G4 G5 G6

Figure S2 – related to Figure 2

(A) Group fold change (GFC) heat map and hierarchical clustering for each sample and the gene modules identified by $CoCena^2$ analysis.

(B) Agglomerative hierarchical clustering of the samples according to the GFC. Top plots present the clustering statistics (within cluster sum of squares and high average silhouette width scores) used for the generation of the six data-driven $CoCena^2$ sample groups G1-G6, which are plotted in the dendrogram plot.

(C) Heat map presenting summary statistics of clinical parameters for COVID-19 patients grouped according to the $CoCena^2$ sample groups G1-G5 Presented are scaled values of the mean value of the parameters age/blood cell counts/SOFA score/Pneumonia Index/Charlson score, and prevalence of the comorbidity/death (outcome)/male (sex)/immune classification (intermediate/dysregulation/MAS). Statistical differences were estimated among the groups via the one-sided Anova test or Fisher test, for numeric or categorical values respectively. (*,** p-value < 0.05, 0.01 respectively)

(D) PCA plot depicting relationship of all samples based on dynamic gene expression of all genes. Coloring based on the six data-driven $CoCena^2$ sample groups G1-G6.

(E) Cibersort cell type deconvolution at cell subset level. Grouping based on the six data-driven $CoCena^2$ sample groups G1-G6.

(F) Flow cytometry analysis, number of lymphocytes (upper) and neutrophils (lower) per µl of blood. Grouping based on the six data-driven $CoCena^2$ sample groups G1-G6.

(G) Heat map of DE and top 20 most variable transcription factors, epigenetic regulators, surface and & secreted proteins.

Fig. S3

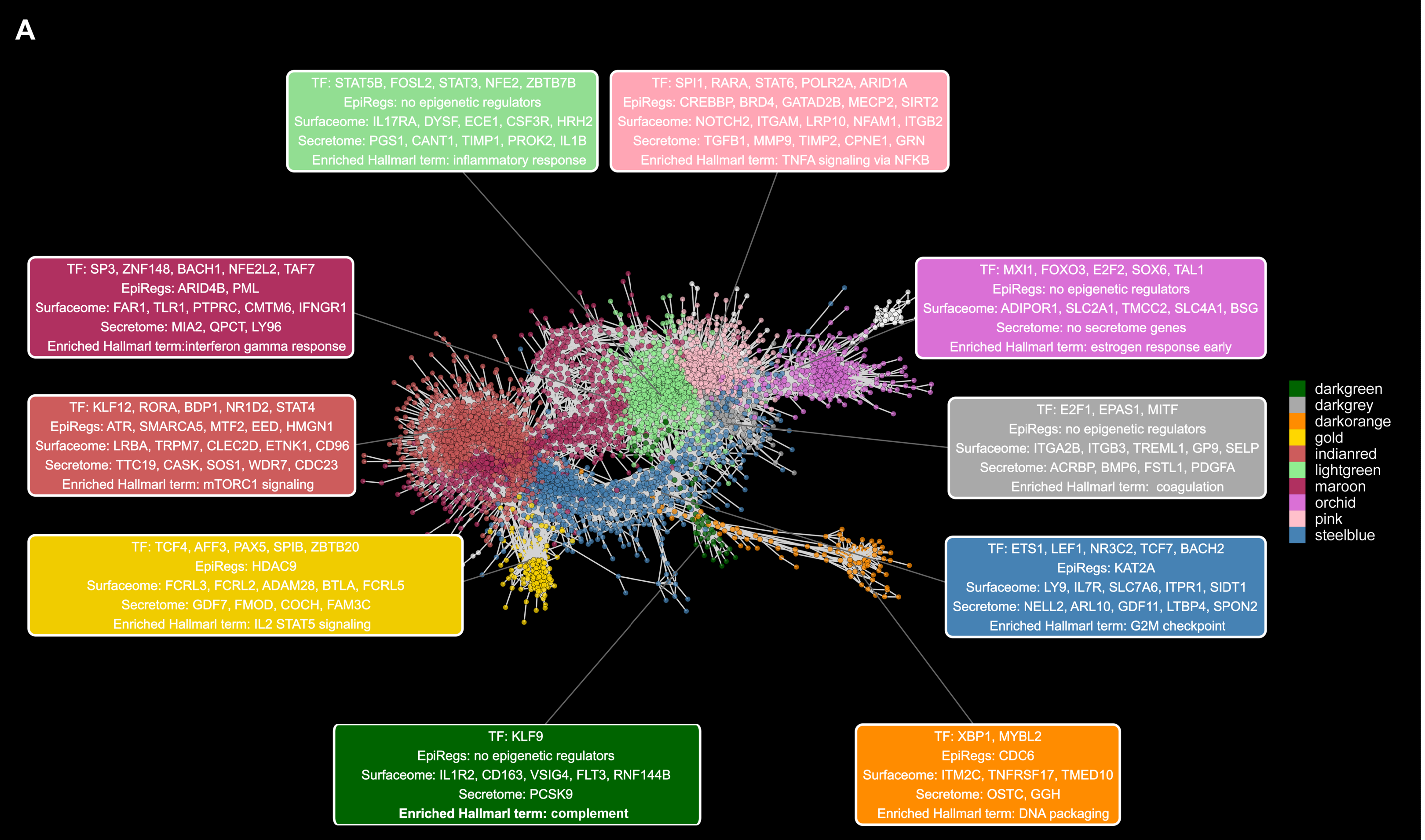

A
TF: STAT5B, FOSL2, STAT3, NFE2, ZBTB7B
EpiRegs: no epigenetic regulators
Surfaceome: IL17RA, DYSF, ECE1, CSF3R, HRH2
Secretome: PGS1, CANT1, TIMP1, PROK2, IL1B
Enriched Hallmarl term: inflammatory response
TF: SPI1, RARA, STAT6, POLR2A, ARID1A
EpiRegs: CREBBP, BRD4, GATAD2B, MECP2, SIRT2
Surfaceome: NOTCH2, ITGAM, LRP10, NFAM1, ITGB2
Secretome: TGFB1, MMP9, TIMP2, CPNE1, GRN
Enriched Hallmarl term: TNFA signaling via NFKB
TF: SP3, ZNF148, BACH1, NFE2L2, TAF7
EpiRegs: ARID4B, PML
Surfaceome: FAR1, TLR1, PTPRC, CMTM6, IFNGR1
Secretome: MIA2, QPCT, LY96
Enriched Hallmarl term:interferon gamma response
TF: MXI1, FOXO3, E2F2, SOX6, TAL1
EpiRegs: no epigenetic regulators
Surfaceome: ADIPOR1, SLC2A1, TMCC2, SLC4A1, BSG
Secretome: no secretome genes
Enriched Hallmarl term: estrogen response early
TF: KLF12, RORA, BDP1, NR1D2, STAT4
EpiRegs: ATR, SMARCA5, MTF2, EED, HMGN1
Surfaceome: LRBA, TRPM7, CLEC2D, ETNK1, CD96
Secretome: TTC19, CASK, SOS1, WDR7, CDC23
Enriched Hallmarl term: mTORC1 signaling
TF: E2F1, EPAS1, MITF
EpiRegs: no epigenetic regulators
Surfaceome: ITGA2B, ITGB3, TREML1, GP9, SELP
Secretome: ACRBP, BMP6, FSTL1, PDGFA
Enriched Hallmarl term: coagulation
darkgreen
darkgrey
darkorange
gold
indianred
lightgreen
maroon
orchid
pink
steelblue
TF: TCF4, AFF3, PAX5, SPIB, ZBTB20
EpiRegs: HDAC9
Surfaceome: FCRL3, FCRL2, ADAM28, BTLA, FCRL5
Secretome: GDF7, FMOD, COCH, FAM3C
Enriched Hallmarl term: IL2 STAT5 signaling
TF: ETS1, LEF1, NR3C2, TCF7, BACH2
EpiRegs: KAT2A
Surfaceome: LY9, IL7R, SLC7A6, ITPR1, SIDT1
Secretome: NELL2, ARL10, GDF11, LTBP4, SPON2
Enriched Hallmarl term: G2M checkpoint
TF: KLF9
EpiRegs: no epigenetic regulators
Surfaceome: IL1R2, CD163, VSIG4, FLT3, RNF144B
Secretome: PCSK9
Enriched Hallmarl term: complement
TF: XBP1, MYBL2
EpiRegs: CDC6
Surfaceome: ITM2C, TNFRSF17, TMED10
Secretome: OSTC, GGH
Enriched Hallmarl term: DNA packaging

Figure S3 – related to Figure 2

(A) Visualization of the COVID-19 CoCena^2 network. Nodes are genes and edges represent co-expressed genes. Additional module information is displayed by module-colored labels. Labels include information about top-connected transcription factors (TFs), epigenetic regulators, surface & secreted markers as well as representative Hallmark terms.

Fig. S4

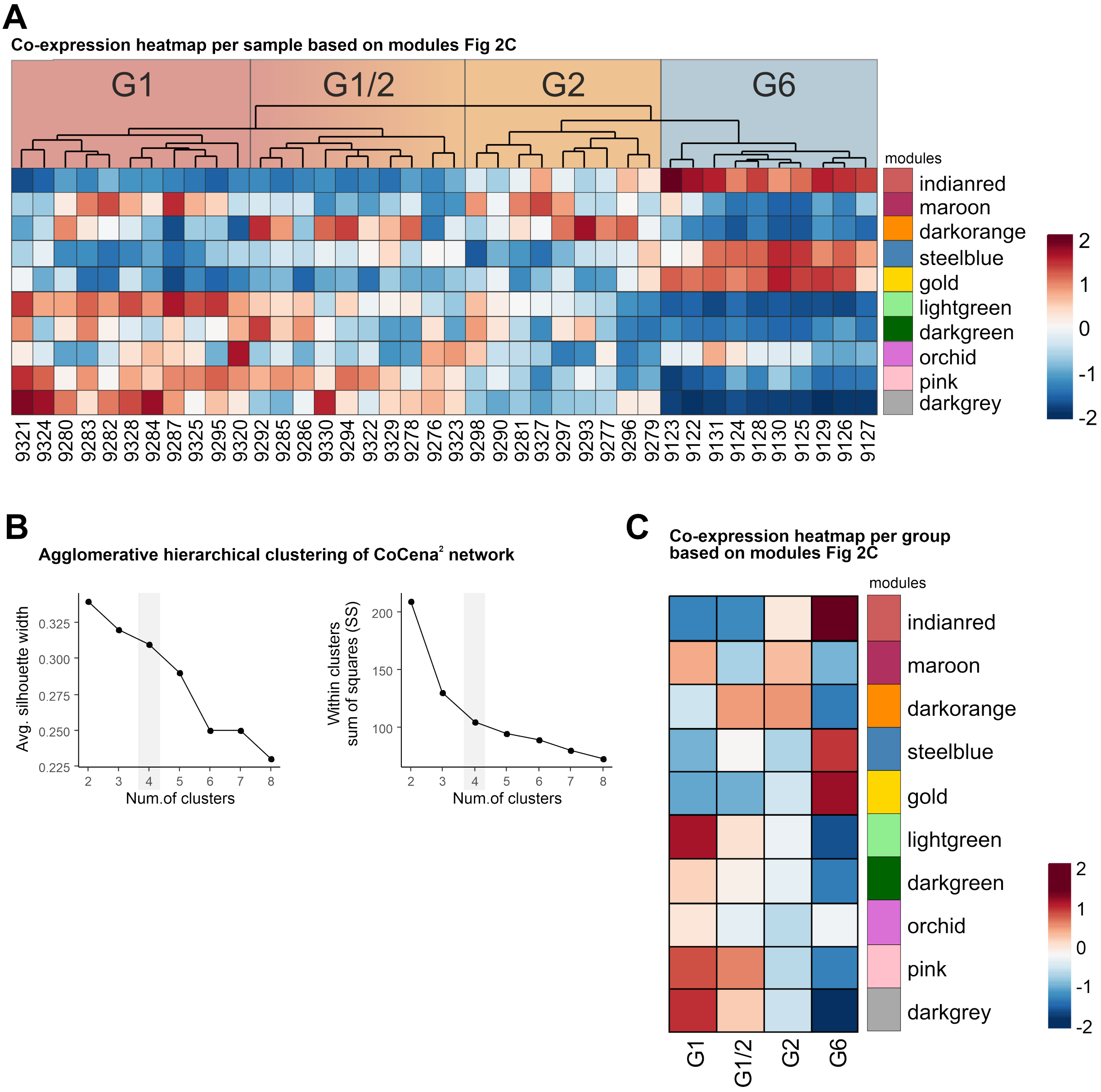
A
Co-expression heatmap per sample based on modules Fig 2C
G1
G1/2
G2
G6
modules
indianred
maroon
darkorange
steelblue
gold
lightgreen
darkgreen
orchid
pink
darkgrey
2
1
0
-1
-2
9321 9324 9280 9283 9282 9328 9284 9287 9325 9295 9320 9292 9285 9286 9330 9294 9322 9329 9278 9276 9323 9298 9290 9281 9327 9297 9293 9277 9296 9279 9123 9122 9131 9124 9128 9130 9125 9129 9126 9127
B
Agglomerative hierarchical clustering of CoCena² network
Avg. silhouette width
0.325
0.300
0.275
0.250
0.225
2 3 4 5 6 7 8
Num.of clusters
Within clusters sum of squares (SS)
200
150
100
2 3 4 5 6 7 8
Num.of clusters
C
Co-expression heatmap per group based on modules Fig 2C
modules
indianred
maroon
darkorange
steelblue
gold
lightgreen
darkgreen
orchid
pink
darkgrey
2
1
0
-1
-2
G1
G1/2
G2
G6

Figure S4 – related to Figure 2

(A) Heat map of mean group fold changes (GFCs) of the $CoCena^2$ whole blood modules in the second COVID-19 cohort for each sample. Patients are clusters by the mean GFC module expression. Severity patterns found in the whole blood $CoCena^2$ network were identified and patients groups were labeled accordingly (G1-G6).

(B) Agglomerative hierarchical clustering of the samples according to the GFC. Top plots present the clustering statistics (within cluster sum of squares and high average silhouette width scores) used for the generation of the six data-driven $CoCena^2$ sample groups G1-G6, which are plotted in the dendrogram plot.

(C) GFC heat map and hierarchical clustering for the four identified sample groups and the gene modules identified with $CoCena^2$ analysis.

Fig. S5

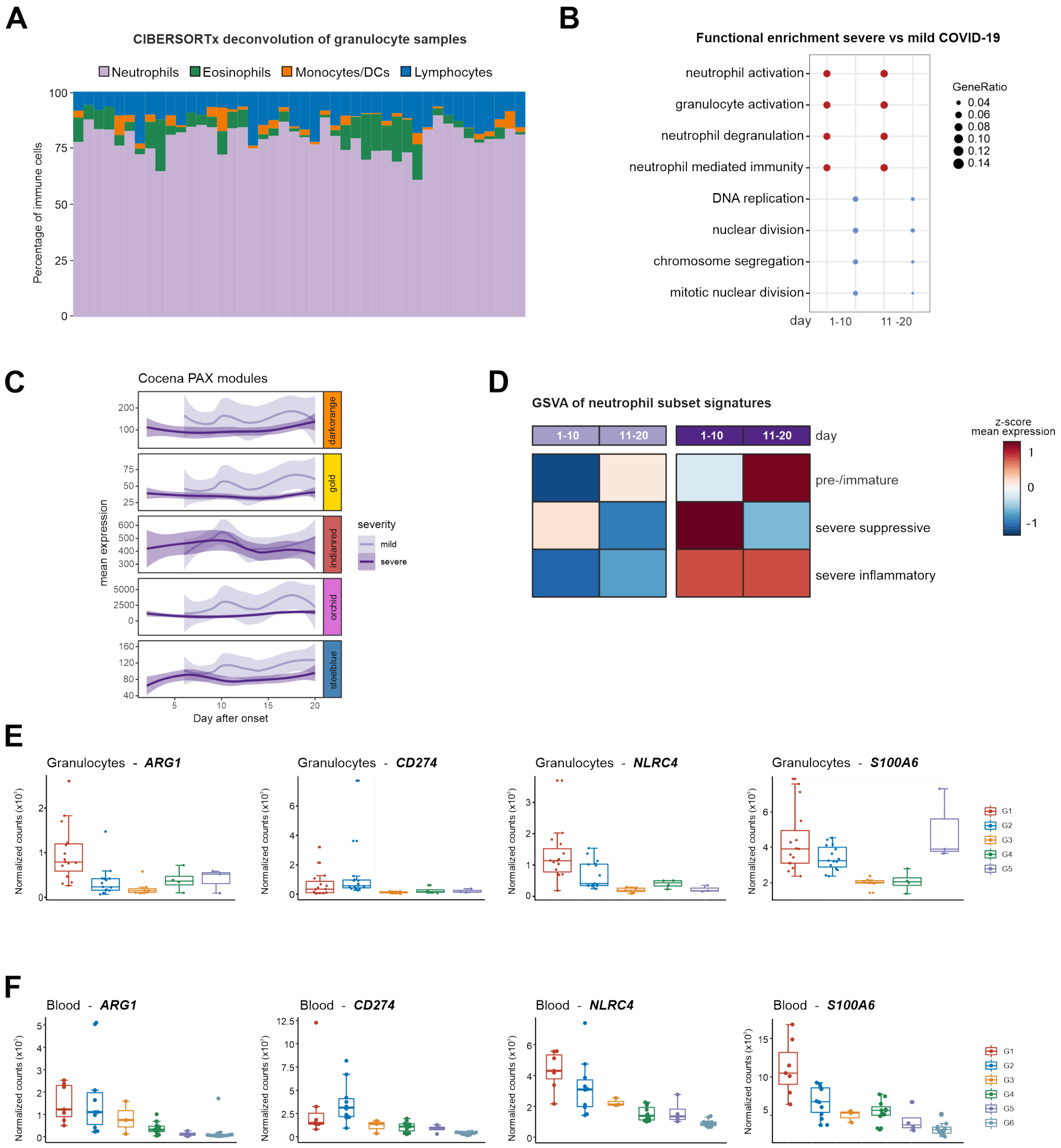
A
CIBERSORTx deconvolution of granulocyte samples
Neutrophils
Eosinophils
Monocytes/DCs
Lymphocytes
Percentage of immune cells
100
75
50
25
0
B
Functional enrichment severe vs mild COVID-19
neutrophil activation
granulocyte activation
neutrophil degranulation
neutrophil mediated immunity
DNA replication
nuclear division
chromosome segregation
mitotic nuclear division
day
1-10
11 -20
GeneRatio
0.04
0.06
0.08
0.10
0.12
0.14
C
Cocena PAX modules
darkorange
gold
indianred
orchid
steelblue
mean expression
Day after onset
severity
mild
severe
D
GSVA of neutrophil subset signatures
1-10
11-20
1-10
11-20
day
pre-/immature
severe suppressive
severe inflammatory
z-score
mean expression
1
0
-1
E
Granulocytes - ARG1
Granulocytes - CD274
Granulocytes - NLRC4
Granulocytes - S100A6
Normalized counts
G1
G2
G3
G4
G5
F
Blood - ARG1
Blood - CD274
Blood - NLRC4
Blood - S100A6
Normalized counts
G1
G2
G3
G4
G5
G6

Figure S5 – related to Figure 3

(**A**) Cibersort computational deconvolution of the 44 granulocyte samples used in Figure 3. The LM22 reference signature was used.

(**B**) Functional enrichment analysis of the DEGs between severe and mild COVID-19 patients by GOEA.

(**C**) Mean of group fold changes (GFCs) of the modules darkorange, gold, indianred, orchid and steelblue in the granulocyte samples of mild (light purple) and severe (purple) COVID-19 cases over time.

(**D**) GSVA of single-cell neutrophil signatures (*34*). Samples are ordered by COVID-19 severity status and days after disease onset.

(**E**) Box plots of *ARG1*, *CD274*, *NLRC4* and *S100A8* in granulocytes grouped by G1-G5.

(**F**) Box plot of *ARG1*, *CD274*, *NLRC4* and *S100A8* in whole blood grouped by G1-G6.

Fig. S6

A

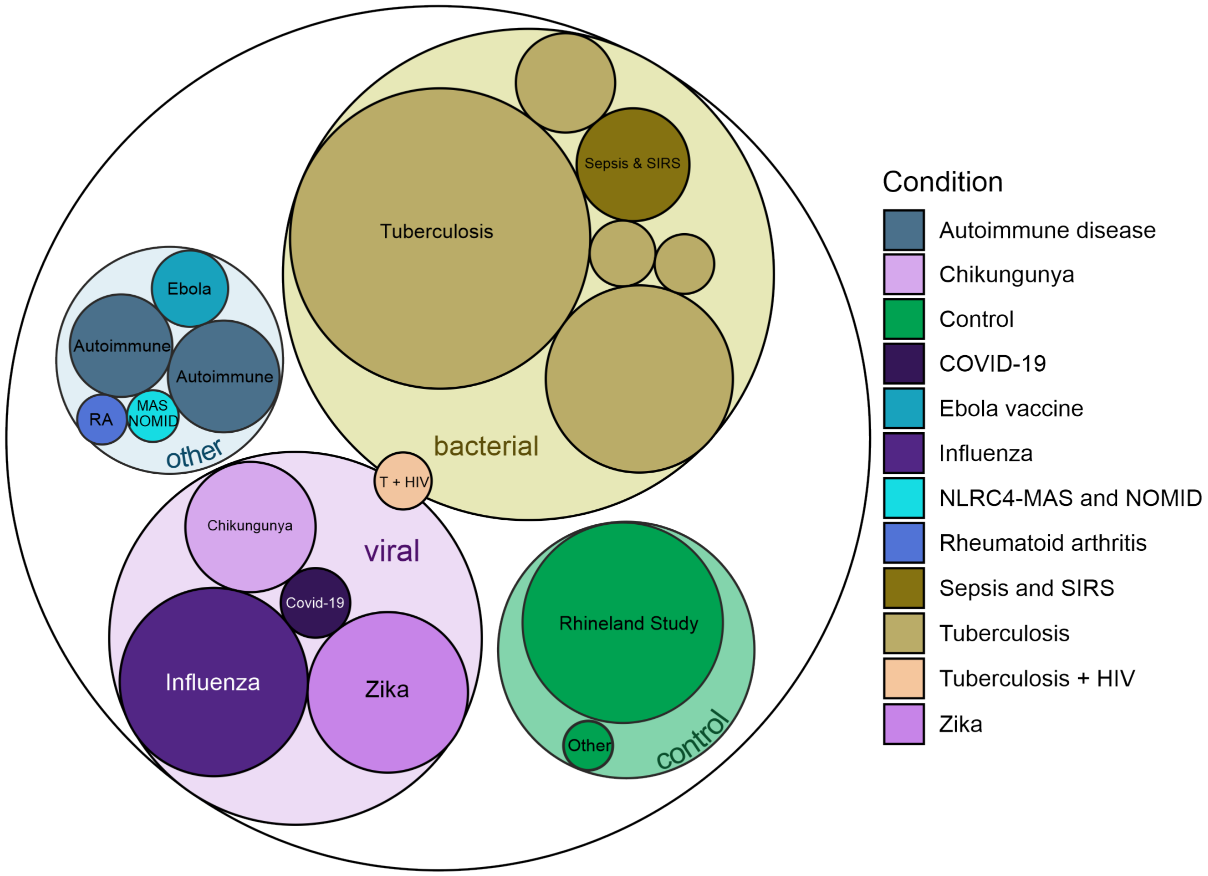

Tuberculosis
Sepsis & SIRS
bacterial
T + HIV
Ebola
Autoimmune
Autoimmune
RA
MAS
NOMID
other
Chikungunya
viral
Covid-19
Influenza
Zika
Rhineland Study
Other
control
Condition
Autoimmune disease
Chikungunya
Control
COVID-19
Ebola vaccine
Influenza
NLRC4-MAS and NOMID
Rheumatoid arthritis
Sepsis and SIRS
Tuberculosis
Tuberculosis + HIV
Zika

Figure S6 – related to Figure 4

(A) Overview of the composition of the integrated dataset comprising 3,176 samples: 39 COIVD-19 samples and 3,137 other conditions and controls.

Fig. S7

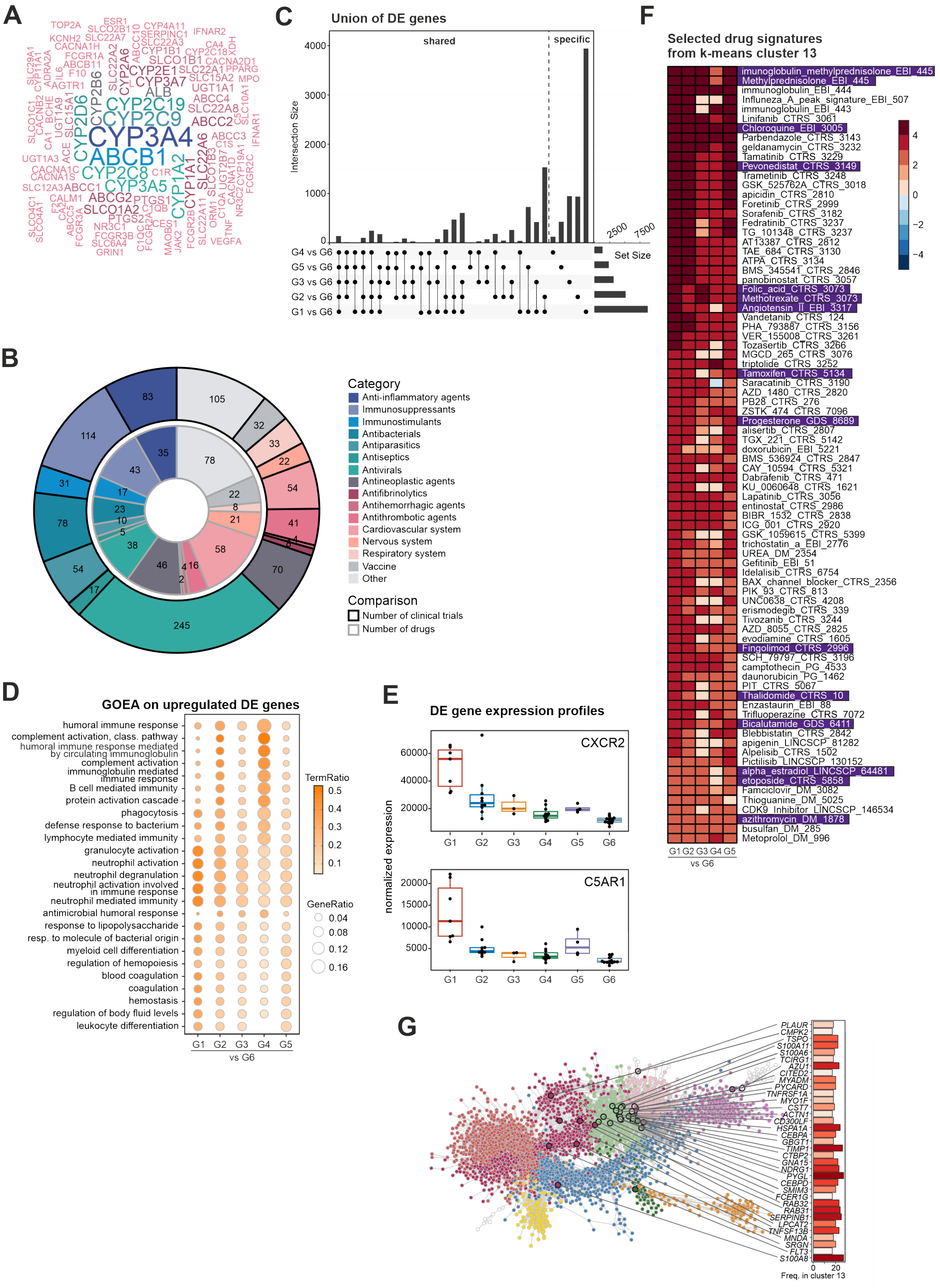

A
CYP3A4
ABCB1
CYP2C19
CYP2C9
CYP2C8
CYP3A5
CYP2D6
CYP1A2
B
Category
Anti-inflammatory agents
Immunosuppressants
Immunostimulants
Antibacterials
Antiparasitics
Antiseptics
Antivirals
Antineoplastic agents
Antifibrinolytics
Antihemorrhagic agents
Antithrombotic agents
Cardiovascular system
Nervous system
Respiratory system
Vaccine
Other
Comparison
Number of clinical trials
Number of drugs
C
Union of DE genes
shared
specific
Intersection Size
Set Size
G4 vs G6
G5 vs G6
G3 vs G6
G2 vs G6
G1 vs G6
D
GOEA on upregulated DE genes
humoral immune response
complement activation, class. pathway
humoral immune response mediated by circulating immunoglobulin
complement activation
immunoglobulin mediated immune response
B cell mediated immunity
protein activation cascade
phagocytosis
defense response to bacterium
lymphocyte mediated immunity
granulocyte activation
neutrophil activation
neutrophil degranulation
neutrophil activation involved in immune response
neutrophil mediated immunity
antimicrobial humoral response
response to lipopolysaccharide
resp. to molecule of bacterial origin
myeloid cell differentiation
regulation of hemopoiesis
blood coagulation
coagulation
hemostasis
regulation of body fluid levels
leukocyte differentiation
TermRatio
GeneRatio
G1 G2 G3 G4 G5
vs G6
E
DE gene expression profiles
CXCR2
C5AR1
normalized expression
G1 G2 G3 G4 G5 G6
F
Selected drug signatures from k-means cluster 13
imunoglobulin_methylprednisolone_EBI_445
Methylprednisolone_EBI_445
immunoglobulin_EBI_444
Influneza_A_peak_signature_EBI_507
immunoglobulin_EBI_443
Linifanib_CTRS_3061
Chloroquine_EBI_3005
Parbendazole_CTRS_3143
geldanamycin_CTRS_3232
Tamatinib_CTRS_3229
Pevonedistat_CTRS_3149
Trametinib_CTRS_3248
GSK_525762A_CTRS_3018
apicidin_CTRS_2810
Foretinib_CTRS_2999
Sorafenib_CTRS_3182
Fedratinib_CTRS_3237
TG_101348_CTRS_3237
AT13387_CTRS_2812
TAE_684_CTRS_3130
ATPA_CTRS_3134
BMS_345541_CTRS_2846
panobinostat_CTRS_3057
Folic_acid_CTRS_3073
Methotrexate_CTRS_3073
Angiotensin_II_EBI_3317
Vandetanib_CTRS_124
PHA_793887_CTRS_3156
VER_155008_CTRS_3261
Tozasertib_CTRS_3266
MGCD_265_CTRS_3076
triptolide_CTRS_3252
Tamoxifen_CTRS_5134
Saracatinib_CTRS_3190
AZD_1480_CTRS_2820
PB28_CTRS_276
ZSTK_474_CTRS_7096
Progesterone_GDS_8689
aliertib_CTRS_2807
TGX_221_CTRS_5142
doxorubicin_EBI_5221
BMS_536924_CTRS_2847
CAY_10594_CTRS_5321
Dabrafenib_CTRS_471
KU_0060648_CTRS_1621
Lapatinib_CTRS_3056
entinostat_CTRS_2986
BIBR_1532_CTRS_2838
ICG_001_CTRS_2920
GSK_1059615_CTRS_5399
trichostatin_a_EBI_2776
UREA_DM_2354
Gefitinib_EBI_51
Idelalisib_CTRS_6754
BAX_channel_blocker_CTRS_2356
PIK_93_CTRS_813
UNC0638_CTRS_4208
erismodegib_CTRS_339
Tivozanib_CTRS_3244
AZD_8055_CTRS_2825
evodiamine_CTRS_1605
Fingolimod_CTRS_2996
SCH_79797_CTRS_3196
camptothecin_PG_4533
daunorubicin_PG_1462
PIT_CTRS_5067
Thalidomide_CTRS_10
Enzastaurin_EBI_88
Trifluoperazine_CTRS_7072
Bicalutamide_GDS_6411
Blebbistatin_CTRS_2842
apigenin_LINCSCP_81282
Alpelisib_CTRS_1502
Pictilisib_LINCSCP_130152
alpha_estradiol_LINCSCP_64481
etoposide_CTRS_5858
Famciclovir_DM_3082
Thioguanine_DM_5025
CDK9_Inhibitor_LINCSCP_146534
azithromycin_DM_1878
busulfan_DM_285
Metoprolol_DM_996
G1 G2 G3 G4 G5
vs G6
G
PLAUR
CMPK2
TSPO
S100A11
S100A6
TCIRG1
AZU1
CITED2
MYADM
PYCARD
TNFRSF1A
MYO1F
CST7
ACTN1
CD300LF
HSPA1A
CEBPA
GBGT1
TIMP1
CTBP2
GNA15
NDRG1
PYGL
CEBPD
SMIM3
FCER1G
RAB32
RAB31
SERPINB1
LPCAT2
TNFSF13B
MNDA
SRGN
FLT3
S100A8
Freq. in cluster 13

Figure S7 – related to Figure 5

(A) Word cloud of the target genes of drugs currently investigated for the treatment of COVID-19 patients. Increasing frequency is represented by increasing size with min. frequency = 41 and max. frequency = 97.

(B) Overview of drugs currently used, investigated or recommended for the treatment of COVID-19 patients. The inner circle represents the number of drugs for the representative drug categories, the outer circle represents the number of clinical trials of drugs for the respective drug categories.

(C) Differentially expressed genes (FC>|2|, FDR-adj. p-value <0.05) of comparisons between groups G1-5 vs G6. Vertical bar plots indicate the number of group-specific differentially expressed genes (DEGs, right) and genes shared by several groups (left), whereby the contributing groups are indicated as connected dots (bottom). Horizontal bar plots visualize the size of DEGs per group.

(D) Gene ontology enrichment analysis of upregulated DEGs obtained for each comparison G1-G5 vs G6. Visualized are significant enrichments (adj. p-value<0.05, q-value<0.05) for the union of top 10 terms per comparison. Term ratio indicates the ratio of DEGs matching the term and the total gene number of that term.

(E) Box plots of normalized expression of selected DEGs, upregulated in at least one comparison.

(F) Display of selected drug signatures from k-means cluster 13 from Fig. 5C showing high ΔNES scores throughout all patient groups compared to G6.

(G) Visualization of recurring target genes in the G1 vs G6 comparison of cluster 13 signatures and their frequency mapped onto the CoCena^2 network

Fig. S8

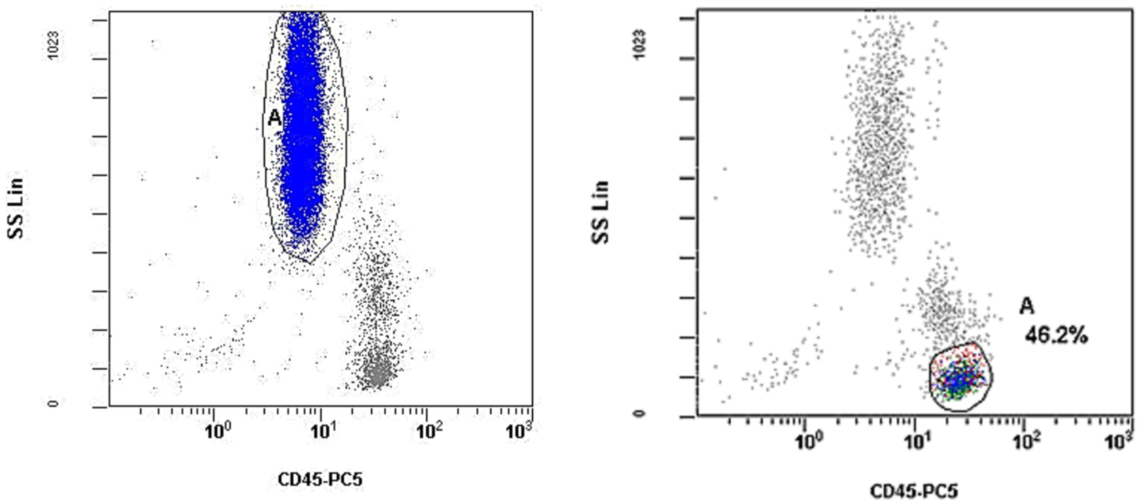

Figure S8 – related to methods gating

(A) Gating to identify neutrophils and lymphocytes using the characteristic sideward scattering of CD45-positive cells.
